# Synthesis of clathrate cerium superhydride CeH_9_ at 80-100 GPa with atomic hydrogen sublattice

**DOI:** 10.1038/s41467-019-12326-y

**Published:** 2019-10-01

**Authors:** Nilesh P. Salke, M. Mahdi Davari Esfahani, Youjun Zhang, Ivan A. Kruglov, Jianshi Zhou, Yaguo Wang, Eran Greenberg, Vitali B. Prakapenka, Jin Liu, Artem R. Oganov, Jung-Fu Lin

**Affiliations:** 1 Center for High Pressure Science & Technology Advanced Research (HPSTAR), 100094 Beijing, China; 2 Department of Geosciences, Center for Materials by Design, and Institute for Advanced Computational Science, State University of New York, Stony Brook, New York, NY 11794-2100 USA; 3 Institute of Atomic and Molecular Physics, Sichuan University, 610065 Chengdu, China; 4 Department of Problems of Physics and Energetics, Moscow Institute of Physics and Technology, 9 Institutskiy Lane, Dolgoprudny City, Moscow Region 141700 Russia; 5 Dukhov Research Institute of Automatics (VNIIA), Moscow, 127055 Russia; 6 Department of Mechanical Engineering, The University of Texas at Austin, Austin, TX 78712 USA; 7 Center for Advanced Radiation Sources, University of Chicago, Chicago, 60637 IL USA; 8 Skolkovo Institute of Science and Technology, Skolkovo Innovation Center, 3 Nobel Street, Moscow, 143026 Russia; 9 International Center for Materials Design, Northwestern Polytechnical University, 710072 Xi’an, China; 10 Department of Geological Sciences, The University of Texas at Austin, Austin, TX 78712 USA

**Keywords:** Superconductors, Electronic properties and materials, Phase transitions and critical phenomena

## Abstract

Hydrogen-rich superhydrides are believed to be very promising high-*T*_c_ superconductors. Recent experiments discovered superhydrides at very high pressures, e.g. FeH_5_ at 130 GPa and LaH_10_ at 170 GPa. With the motivation of discovering new hydrogen-rich high-*T*_*c*_ superconductors at lowest possible pressure, here we report the prediction and experimental synthesis of cerium superhydride CeH_9_ at 80–100 GPa in the laser-heated diamond anvil cell coupled with synchrotron X-ray diffraction. Ab initio calculations were carried out to evaluate the detailed chemistry of the Ce-H system and to understand the structure, stability and superconductivity of CeH_9_. CeH_9_ crystallizes in a *P6*_*3*_*/mmc* clathrate structure with a very dense 3-dimensional atomic hydrogen sublattice at 100 GPa. These findings shed a significant light on the search for superhydrides in close similarity with atomic hydrogen within a feasible pressure range. Discovery of superhydride CeH_9_ provides a practical platform to further investigate and understand conventional superconductivity in hydrogen rich superhydrides.

## Abstract (web summary)

Hydrogen-rich superhydrides are promising high-temperature superconductors which have been observed only at pressures above 170 GPa. Here the authors show that CeH_9_ can be synthesized at 80-100 GPa with laser heating, and is characterized by a clathrate structure with a dense 3-dimensional atomic hydrogen sublattice.

## Introduction

Metallization of hydrogen under high pressure has been a topic of great scientific interest in the past few decades mainly due to expectations of room-temperature superconductivity^1–7^. Hydrogen is expected to become metallic under high pressure above 400 GPa^7–9^. But achieving such pressures and verifying superconductivity are very challenging in diamond anvil cell (DAC) experiments, mainly due to diamond failure and lack of a reliable probe on the tiny sample volumes at such high pressures. Alternatively, hydrogen-rich hydrides can also be expected to achieve high-*T*_c_ superconductivity perhaps at a much lower pressure than that of required for metallic hydrogen^10–12^. Both hydrides and metallic hydrogen are expected to be conventional superconductors. High phonon frequency, strong electron–phonon coupling, and high density of states at the Fermi level are the essential conditions for superconductivity with Cooper pairs mediated by electron–phonon interaction^13^. Hydrides may satisfy all these conditions as the low mass of hydrogen results in high phonon frequency; covalent bonding is favourable for strong electron–phonon coupling; metallization under high pressure can result in high electronic density of states at the Fermi level^10^. Within this view the remarkable prediction and experimental confirmation of superconductivity at a record high *T*_c_ of 203 K under pressure of 150 GPa in H_3_S makes sense^14,15^. The discovery of superconductivity in H_3_S gave hopes to achieve room-temperature superconductivity in hydrogen-rich systems under high pressure. Recently superconductivity with *T*_c_ of 260 K at 180 GPa and 250 K at 170 GPa were reported for LaH_10_ by two different research groups by electrical conductivity measurement^16,17^. Detecting Meissner effect to confirm the superconductivity has been difficult because signal from extremely small sample could be too weak to be picked up by the state-of-the-art techniques. At such pressure the verification of *T*_c_ becomes a challenging task. A recent study by Drozdov et al. reported *T*_c_ of 250 K in LaH_10_, which  decreases with the application of magnetic field, and the isotope effect was also observed^17^. The discovery of superconductivity in LaH_10_ is a milestone in the search of room temperature superconductivity.

Hydrogen readily reacts with most elements to form binary hydrides^18,19^. Several hydrogenic motifs such as H^δ^^−^, H_2_^δ−^, H_3_^−^, H_3_^+^, H_4_^−^, and H_5_^+^, and infinite chains, layers, frameworks were predicted to occur in high-pressure hydrides^20–22^. One-dimensional hydrogen chains and three-dimensional clathrate structures with hydrogen cage were predicted and found to be good candidates for high *T*_c_ superconductivity^20^. Recent theoretical predictions have reported several systems with unusually high hydrogen content, termed as polyhydrides/superhydrides, to become stable under high pressure and to exhibit significantly high *T*_c_ under pressure^22–29^. Notably CaH_6_^24^, MgH_6_^26^, YH_6_^27^_,_ YH_9_^23^, YH_10_^23,28^, LaH_10_^28^, ThH_10_^30^, AcH_10_, and AcH_16_^29^ were predicted to have *T*_c_ above 235 K. Most of the phases with *T*_c_ close to room temperature are predicted to have a clathrate structure with hydrogen forming a cage around metal atom (*M*). In *M*H_6_, *M*H_9_, and *M*H_10_ compounds, metal atoms are located within H_24_, H_29_, and H_32_ cages, respectively^23,24,27,28^. However, it is essential to know the experimental pressure–temperature condition to stabilize a hydride before carrying out the further electrical or magnetic measurement to verify the superconductivity. Recently, a handful of experiments were reported to synthesize new superhydrides under pressure, particularly FeH_5_ at 130 GPa^31^, LaH_10_ at 170 GPa^32^, UH_7_, UH_8_, and UH_9_ above 37 GPa^33^. There were also experimental reports about synthesis of new and unusual hydrides under pressure, such as LiH_6_^34^, NaH_7_^35^, Xe(H_2_)_7_^36^, and HI(H_2_)_13_^37^ with H_2_-like molecular units. Hydrides possessing H_2_-molecular units are not prone to have higher *T*_c_ as they tend to have low densities of states at the Fermi level^23^. Synthesis of FeH_5_ and LaH_10_ without any H_2_-like unit is very intriguing. Interestingly, FeH_5_ crystallized in layered structure consists two-dimensional atomic hydrogen slabs. LaH_10_ has three-dimensional clathrate structure in which La atoms surrounded by hydrogen cage. H_3_S has body-centered cubic structure which can also be visualized as sulfur atom surrounded by three-dimensional hydrogen cage. Nearest H–H distance in FeH_5_ was reported to be ~1.336 Å at ~100 GPa^31^, whereas for LaH_10_ it was ~1.196 Å at ~120 GPa^32^. LaH_10_ was claimed as a closest analogue to solid atomic metallic hydrogen based on nearest H–H distance^32^. However, the pressure required to stabilize FeH_5_ and LaH_10_ phases was 130 and 170 GPa, respectively^31,32^. Synthesis of superhydrides at lower pressures would give an opportunity to further investigate superconductivity in these hydrides with a wide range of probes. Studies on the synthesis path and structure of superhydrides also help to build a deeper understanding of hydride chemistry. Besides superconductivity, hydrides are also very important as hydrogen storage materials for next-generation energy-related applications^38^. Recently, Peng et al.^23^ predicted that hydrogen-rich CeH_9_ with *P*6_3_/*mmc* structure becomes stable at a relatively low pressure of 100 GPa, which by itself is very interesting although their estimated superconducting *T*_c_ was relatively low, <56 K.

We have carefully studied the Ce–H system in order to understand the crystal chemistry and to seek for superconductivity with, possibly, much higher *T*_c_ values. Here we report the successful synthesis of cerium superhydride CeH_9_ at 80-100 GPa with laser heating up to ~2000 K. Using evolutionary variable-composition searches, the whole compositional space of the Ce–H system explored in a single simulation. We predicted phase stability and superconducting properties of high-pressure cerium superhydrides. Rich chemistry of cerium hydrides manifests itself in numerous stable compounds, including the experimentally synthesized CeH_3_ and superhydrides CeH_9_. We have carried out a direct elemental reaction between cerium and hydrogen using a laser-heated DAC coupled with synchrotron X-ray diffraction (XRD). It is found that heating plays an essential role in the formation of Ce–H phases at high pressures. Analysis of XRD results in combination with ab initio calculations shows that CeH_9_ crystallizes in a clathrate structure with space group *P6*_*3*_*/mmc* at 80-100 GPa after laser heating. Each cerium atom is enclosed within a cage of H_29_ in which hydrogen atoms are bonded covalently. Besides this, a previously unknown $Pm\bar 3n$ structured CeH_3_ (*β*-UH_3_ type^39^) was synthesized at 36 GPa with laser heating. The detailed first-principles investigation of stability, structural, electronic, and superconducting properties of experimentally synthesized hydrogen-rich phase was carried out. We studied, specifically electron–phonon interaction of *P6*_*3*_*/mmc*-CeH_9_ and predicted that the CeH_9_ is a high temperature superconductor with *T*_c_ = 105–117 K at 200 GPa.

## Results

### Synthesis of various Ce–H phases

**Fig. 1:**
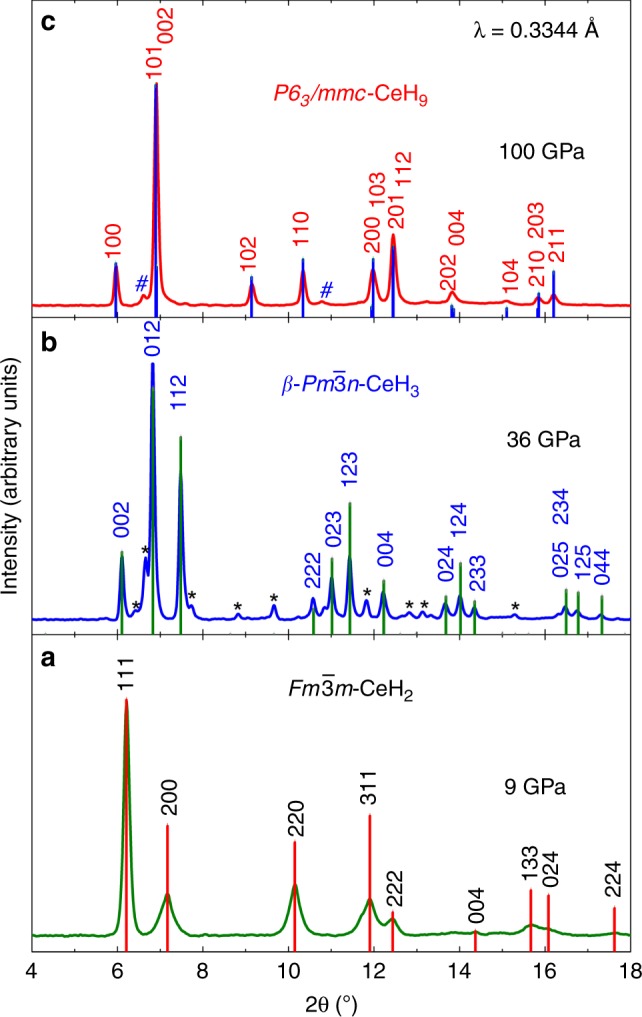
Representative integrated XRD patterns of high-pressure phases in Ce–H system up to 100 GPa. Typical XRD patterns of **a** CeH_2_, **b** CeH_3_, and **c** CeH_9_ obtained at 9, 36, and 100 GPa of pressure respectively. Vertical lines indicate the indexing with calculated intensity for respective crystal structure. CeH_2_, CeH_3_, and CeH_9_ crystallize in space group $Fm\bar 3m$, $Pm\bar 3n$ (*β*-UH_3_ type) and *P6*_*3*_*/mmc*, respectively. Unidentified weak peaks in **b** and **c** are marked with black asterik and blue hash symbols, respectively. These additional peaks could not be identified or indexed with any of the known or predicted phases of Ce^68^ or CeH_*x*_^23^, as well as cerium oxides^69,70^

In our experiment, various phases of the Ce–H system such as CeH_*x*_ (*x* = 2, 2.5, 3, and 9) were synthesized successfully at high pressures. Initially, the cerium sample and hydrogen gas were loaded into the sample chamber of the DAC and were kept at 9 GPa. A small piece of gold also was loaded along with sample to calibrate pressure. At 9 GPa and ambient temperature, we found that cerium and hydrogen reacted, which resulted in the formation of a cerium hydride compound as shown by the XRD pattern in Fig. 1a. The corresponding XRD image is shown in Supplementary Fig. 1a. All the peaks observed at 9 GPa could be indexed with the $Fm\bar 3m$ phase of CeH_2_ (see Supplementary Note 1). The $Fm\bar 3m$ phase of CeH_2_ persisted up to 33 GPa (Supplementary Fig. 1b). Lebail refinements were carried to extract lattice parameters of the CeH_2_ phase (Supplementary Fig. 1c). The lattice parameters of CeH_2_ at 9 and 33 GPa were determined as *a* = 5.370(1) and *a* = 5.011(2) Å respectively. Pressure dependence of the unit cell volume of CeH_2_ was fitted with a third-order Birch–Murnaghan equation of state (EOS) which yielded the fitting parameters such as unit cell volume at zero pressure *V*_0_ = 44(1) Å^3^ per f.u., bulk modulus *K*_0_ = 45(6) GPa and first pressure derivative of bulk modulus *K*_0_′ = 4 (fixed) (Supplementary Fig. 1d). Pressurization of CeH_2_ up to 33 GPa did not result in any changes in crystal structure. However, microsecond pulsed laser heating of ~2000 K carried at 33 GPa resulted in obvious structural changes (Supplementary Fig. 2a). The XRD pattern at 36 GPa obtained after laser heating is shown in Fig. 1b, and the corresponding XRD image is shown in Supplementary Fig. 2b. The integrated XRD pattern at 36 GPa was found to be of cubic CeH_3_ with $Pm\bar 3n$ isomorphous to *β*-UH_3_ $(\beta \mbox {-}Pm\bar 3n)$^39^. This high-pressure phase of CeH_3_ with $\beta \mbox {-}Pm\bar 3n$ structure has also been predicted in our evolutionary searches to be the energetically favourable phase below 10 GPa and is 32 meV/atom higher in enthalpy than the most favourable CeH_3_ at the synthesized pressure of 36 GPa (Supplementary Fig. 3). However, inclusion of spin–orbit coupling (SOC) and magnetism changes the enthalpy difference of CeH_3_ phases by some 0.09 eV per atom. While these calculations affect predicted phase stability range, our experimental results confirm the existence of the predicted phase. To the best of our knowledge, *β*-UH_3_ type $Pm\bar 3n\mbox {-}{\mathrm{CeH}}_{\mathrm{3}}$ is being reported for the first time here. The experimental lattice parameters of the $\beta \mbox {-}Pm\bar 3n$ phase at 36 GPa are *a* = 6.2788(3) Å. In $\beta \mbox {-}Pm\bar 3n$ structure of CeH_3_, cerium atoms occupy 2a (0, 0, 0) and 6c (1/4, 0, 1/2) Wyckoff positions^39^. Unfortunately, very low X-ray scattering factor of hydrogen atom did not allow us to determine the exact position of hydrogen atoms in CeH_3_ unit cell from the experimental XRD data. Theoretical calculations yielded the Wyckoff position for the hydrogen atoms as 24 K (0, 0.1580, 0.6935) at 35 GPa with lattice parameter *a* = 6.2471 Å, which is highly consistent with the experimental value of $\beta \mbox {-}Pm\bar 3n\mbox {-}{\mathrm{CeH}}_{\mathrm{3}}$ observed at 36 GPa. The $\beta \mbox {-}Pm\bar 3n$ phase of CeH_3_ proved stable with further compression up to 80 GPa and also sustained laser heating at an intermediate pressure of 60 GPa (Supplementary Fig. 2a), which agrees with our predictions. A third-order Birch–Murnaghan EOS was used to fit the *P*–*V* data of CeH_3_ (see Supplementary Fig. 2d), fitting parameters are *V*_*0*_ = 39.7(4) Å^3^ per f.u., *K*_0_ = 86(4) GPa and *K*_0_′ = 4 (fixed).

**Fig. 2:**
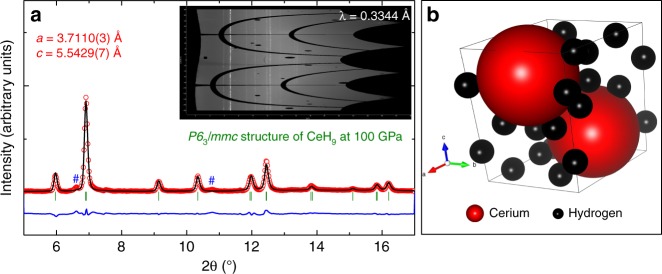
XRD pattern and the result of Rietveld refinement for the hexagonal CeH_9_ in *P6*_*3*_*/mmc* structure. **a** Rietveld refinement plot of powder XRD data at 100 GPa. Red open circles: experimental data of CeH_9_ in *P6*_*3*_*/mmc* structure at 100 GPa; black line: simulated XRD based on the structural model; green vertical lines: Bragg diffraction positions of the structure; blue line: the difference between the simulated and the original XRD. Reliability parameters for the Rietveld refinement are as follows (in %): *R*_p_ = 14.5, *R*_wp_ = 18.4, *R*_Bragg_ = 8.05. Blue hash symbols represent unidentified weak peaks. Inset shows the Pilatus XRD image of corresponding powder XRD pattern with the incident X-ray wavelength of 0.3344 Å. **b** Crystal structure model of *P6*_*3*_*/mmc* structured CeH_9_. Red and black spheres represent cerium and hydrogen atoms, respectively

Several cycles of pulse laser heating with 1 µs pulse width at a repetition rate of 10 kHz for a total heating duration of a few seconds each cycle was used to laser heat the sample assemblage to approximately 1700 K at 80 GPa that resulted in the emergence of new peaks (Supplementary Fig. 4). These new diffraction peaks were indexed to be (101) and (002) of clathrate hexagonal phase of CeH_9_ (Figs. 1c and 2, also see in Supplementary Fig. 4) as found by our evolutionary search. With further pressurization the relative intensity of the (101) and (002) peaks of CeH_9_ phase increased (Supplementary Fig. 4). Although most of the peaks of CeH_3_ phase were present, the intensity of the CeH_9_ peaks became prominent and increased with pressure (Supplementary Fig. 4). Several further cycles of laser heating at 88 GPa (Supplementary Fig. 4) and 98 GPa improved the intensity of the hexagonal CeH_9_ phase, as shown in Fig. 1c. A Rietveld refinement plot for the CeH_9_ phase at 100 GPa is shown in Fig. 2a with the corresponding XRD image as an inset. CeH_9_ crystallizes in the *P6*_*3*_*/mmc* space group with lattice parameters *a* = 3.7110(3) and *c* = 5.5429(7) Å at 100 GPa. Cerium atoms occupy the Wyckoff position 2d (2/3, 1/3, 1/4) in a hexagonal unit cell. Theoretical calculations established positions of hydrogens to be 2b (0, 0, 1/4), 4f (1/3, 2/3, 0.1499), and 12k (0.1565, 0.8435, 0.4404) at 100 GPa. The crystal structure of CeH_9_ is shown in Fig. 2b. The experimentally observed *P6*_*3*_*/mmc* CeH_9_ phase and its structural parameters are perfectly consistent with our calculations. Calculated EOS parameters for CeH_9_ phase are *V*_0_ = 53.4(2) Å^3^ per f.u., *K*_0_ = 80.5(13) GPa and *K*_0_′ = 4 (fixed). Synthesis of the *P6*_*3*_*/mmc*-CeH_9_ phase has been confirmed in two independent experimental runs.

**Fig. 3:**
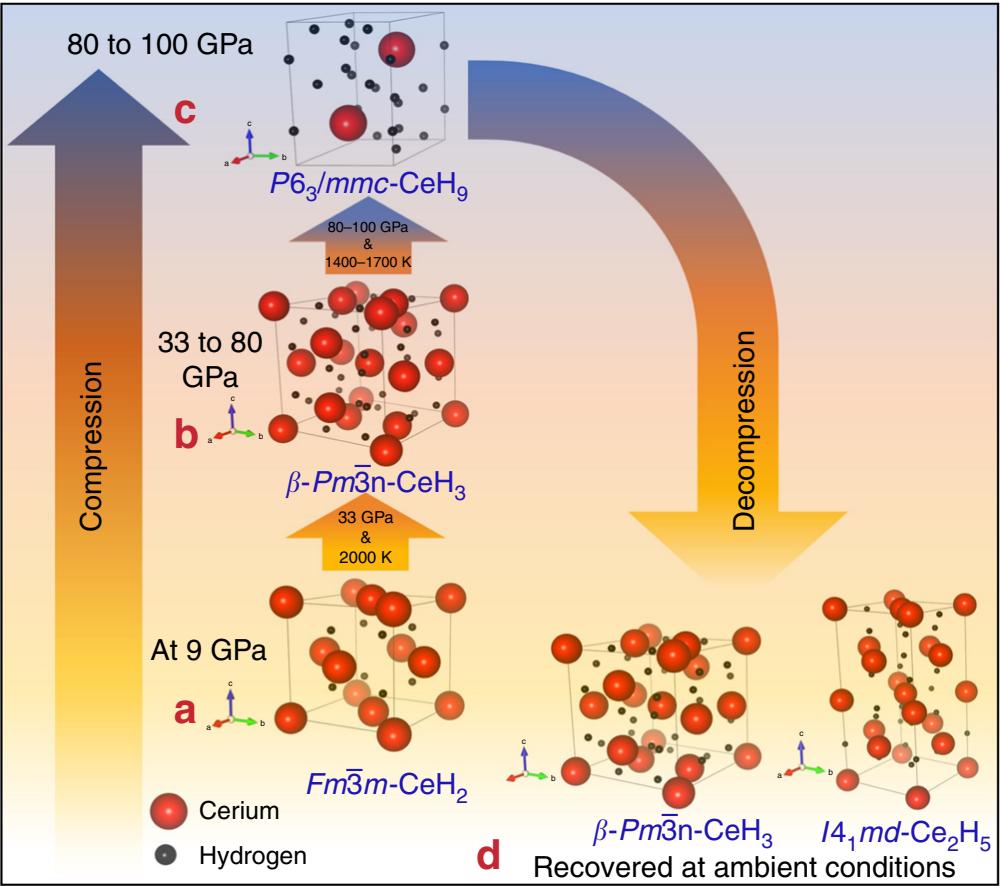
Pressure temperature path for the synthesis and stability of various Ce–H phases. **a** Starting at 9 GPa, cerium reacts with hydrogen to form $Fm\bar 3m\mbox {-}{\mathrm{CeH}}_{\mathrm{2}}$, which remained stable up to 33 GPa. **b** At 33 GPa with laser heating, $Fm\bar 3m\mbox {-}{\mathrm{CeH}}_{\mathrm{2}}$ in H_2_ medium reacted to form $\beta \mbox {-}Pm\bar 3n\mbox {-}{\mathrm{CeH}}_{\mathrm{3}}$. $\beta \mbox {-}Pm\bar 3n\mbox {-}{\mathrm{CeH}}_{\mathrm{3}}$ remained stable up to 80 GPa. **c** Laser heating of $\beta \mbox {-}Pm\bar 3n\mbox {-}{\mathrm{CeH}}_{\mathrm{3}}$ in H_2_ medium at 80-100 GPa resulted in the occurrence of the *P6*_*3*_*/mmc*-CeH_9_ superhydride. The superhydride phase was found to be stable up to the maximum pressure reached in our studies i.e. 100 GPa. **d** After complete decompression, $\beta \mbox {-}Pm\bar 3n \mbox{-}{\mathrm{CeH}}_{\mathrm{3}}$ and $I4_1md\mbox {-}{\mathrm{Ce}}_{\mathrm{2}}{\mathrm{H}}_{\mathrm{5}}$ were recovered at ambient conditions

During the decompression cycle, the CeH_9_ phase was observed to start to become unstable at pressures below 93 GPa and ambient temperature (supplementary Fig. 5). Laser heating was also carried out during the decompression cycle at 79 and 54 GPa. Laser heating in decompression cycle did not result in any changes in XRD pattern. Upon further decompression, the $\beta \mbox {-}Pm\bar 3n\mbox {-}{\mathrm{CeH}}_{\mathrm{3}}$ phase reappeared below 50 GPa (supplementary Fig. 5). Finally, after complete decompression, the $\beta \mbox {-}Pm\bar 3n\mbox {-}{\mathrm{CeH}}_{\mathrm{3}}$ phase was recovered at ambient conditions along with tetragonal Ce_2_H_5_ (Supplementary Fig. 5). The *P*-*T* path for the formation and stability of various Ce–H phases, observed in our experiments, can be seen in Fig. 3. All these findings are repeatable as demonstrated in a separate run of experiment (see Supplementary Note 2 and Supplementary Fig. 6).

### Theoretical calculations and prediction of cerium hydrides

**Fig. 4:**
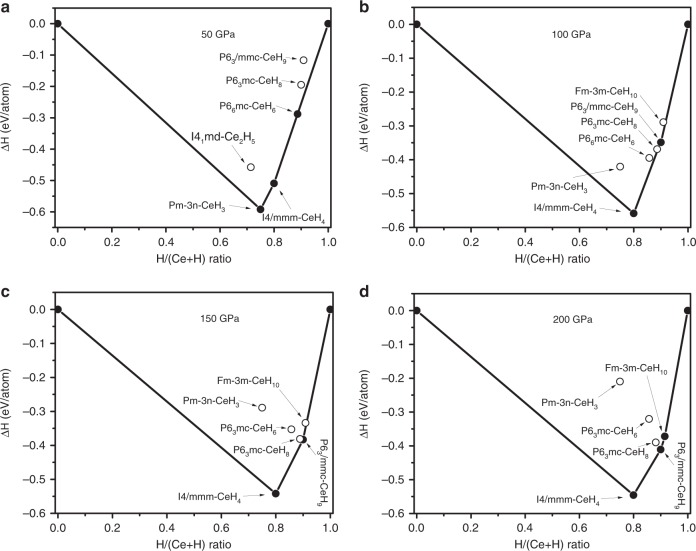
Convex hull diagram of Ce–H system. Predicted formation enthalpy of Ce_1−*x*_ H_*x*_ as a function of H concentration at selected pressures. Open circles above the convex hull show unstable compounds with respect to decomposition into the two adjacent phases on the convex hull, while solid circles show thermodynamically stable compounds

**Fig. 5:**
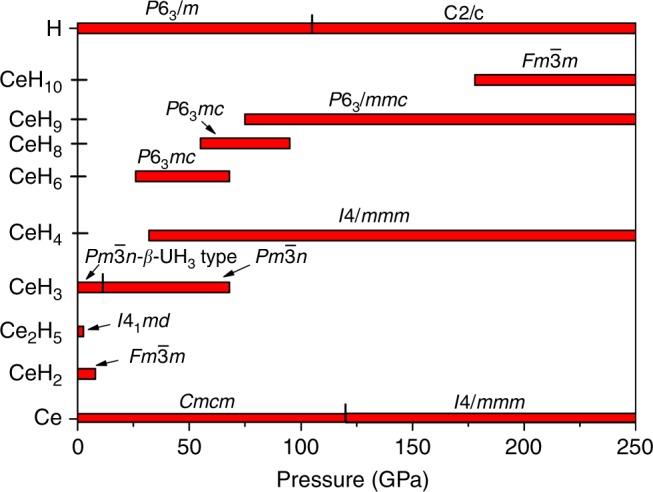
Pressure–composition phase diagram of theoretically predicted stable phases in the Ce–H system at high pressures. Red horizontal bars show the range of stability of each phase; this phase diagram was created on the basis of the evolutionary structure prediction method USPEX. The experimentally discovered *P6*_*3*_*/mmc*-CeH_9_ is predicted to be stable from 78 GPa up to at least 250 GPa.

First-principles calculations were carried out to understand the detailed chemistry of the Ce–H system, dynamical stability, and structural and electronic band structures of experimentally synthesized superhydride phases. We performed variable-composition evolutionary searches at 0, 50, 100, 150, 200, and 250 GPa. The thermodynamic convex hull at different pressures is depicted in Fig. 4. The predicted stable cerium hydrides are shown in the pressure–composition phase diagram in Fig. 5. Several compounds such as *I4/mmm*-CeH_4_, *P6*_*3*_*mc*-CeH_6_, *P6*_*3*_*mc*-CeH_8_, *P6*_*3*_*/mmc*-CeH_9_. and $Fm\bar 3m\mbox {-}{\mathrm{CeH}}_{{\mathrm{10}}}$, were predicted, in addition to finding three known compounds CeH_2_, Ce_2_H_5_, and CeH_3_. High-pressure phase of the CeH_3_ was also predicted with space group $Pm\bar 3n$, as shown in Fig. 5. Because of high concentration of hydrogen in hydrogen-rich hydrides, zero-point  energy (ZPE) might be important in determining the relative stability of hydrogen-rich phases; however, in our previous studies^22,40^, we showed that this quantum effect does not change the topology of the phase diagram, and quantitative effects are just moderate shifts in transition pressures. For example, for GeH_4_ the inclusion of ZPE shifts the transition pressure *Ama2* → *C2/m* from 300 to 278 GPa^40^. Among the stable phases predicted, we have synthesized $Fm\bar 3m\mbox {-}{\mathrm{CeH}}_{\mathrm{2}}$, $I4_1md\mbox {-}{\mathrm{Ce}}_{\mathrm{2}}{\mathrm{H}}_{\mathrm{5}}$ $\beta \mbox {-}Pm\bar 3n\mbox {-}{\mathrm{CeH}}_{\mathrm{3}}$, and $P6_3/{mmc}\mbox {-}{\mathrm{CeH}}_{\mathrm{9}}$. Our pressure–composition phase diagram shows pressure ranges of stability for all the predicted phases along with experimentally known compounds. It clearly shows that higher pressures favour higher hydrogen content compounds, which is consistent with our experiment done at different pressure conditions. Previously known compounds $Fm\bar 3m\mbox {-}{\mathrm{CeH}}_{\mathrm{2}}$ and $I4_1md\mbox {-}{\mathrm{Ce}}_{\mathrm{2}}{\mathrm{H}}_{\mathrm{5}}$ are predicted to be stable only below 8 and 1.5 GPa, respectively. Increase of pressure leads to the formation of *I4/mmm*-CeH_4_ above 32 GPa. CeH_6_ and CeH_8_ are stable in relatively narrow pressure ranges from 26 to 68 GPa, and 55 to 95 GPa, respectively and that is probably why they are not observed in our experiment. *P6*_*3*_*/mmc*-CeH_9_ becomes stable at pressures above 78 GPa, which agrees with our high-pressure experiments where it was synthesized at 80-100 GPa after laser heating. Detailed structural information on the predicted phases can be found in Supplementary Table 1. Among the predicted stable cerium hydrides, we focus on modelling of hydrogen-rich CeH_9_, since a higher hydrogen to metal ratio in hydrides is expected to correlate with higher *T*_c_ superconductivity in hydrogen-rich hydrides^11^.

## Discussion

**Fig. 6:**
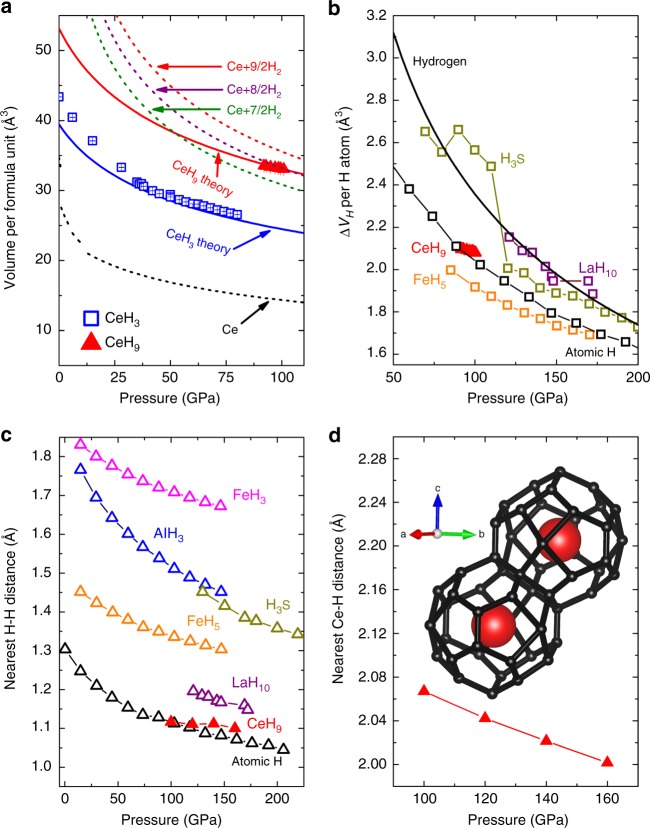
Effect of hydrogen on unit cell volume and nearest neighbour distances in various hydrides at different pressures. **a** Experimentally obtained volume per formula unit for CeH_3_ and CeH_9_ plotted as a function of pressure. Blue open squares and red solid tringles represent experimental data for CeH_3_ and CeH_9_ respectively. Errors in the fitting are also plotted. Theoretical EOS of CeH_3_ and CeH_9_ is plotted as blue and red lines, respectively. Black dashed line represents EOS of cerium metal^68^. Red, purple and green dashed curves represent ideal mixtures of Ce+(9/2)H_2_, Ce+(8/2)H_2_, and Ce+(7/2)H_2_, respectively^68,71^. **b** Volume expansion (Δ*V*_H_) per hydrogen atom plotted against pressure for CeH_9_, FeH_5_^31^, H_3_S^14^, LaH_10_^32^, H^71^, and atomic H^31^ for comparison. Dark yellow, purple, black, and orange open triangle symbol-line represents H_3_S, LaH_10_, atomic H, and FeH_5_, respectively. Red solid triangle symbols represent CeH_9_. Black solid line represents hydrogen. **c** Comparison of the pressure dependence of the nearest H–H distances for CeH_9_, FeH_3_^41^, FeH_5_^31^, AlH_3_^42^, H_3_S^14^, LaH_10_^32^, and atomic H^31^. Magenta, blue, dark yellow, orange, purple, and black open triangle symbol line represents FeH_3_, AlH_3_, H_3_S, FeH_5_, LaH_10_, and atomic H, respectively. Red solid triangle symbol line represents CeH_9_. **d** Nearest Ce–H distance for CeH_9_ as a function of pressure. Inset shows CeH_29_ clathrate cage in *P6*_*3*_*/mmc* structure. [Pressure-dependent experimental data is at 300 K, whereas theoretical data is at 0 K]

Addition of hydrogen in metallic sublattice expands the unit cell volume. In most cases, an increase in volume is proportional to hydrogen content in hydride. In hydrides, expansion of the cell volume with respect to pure metal volume was frequently used to establish the hydrogen content and stoichiometry^32^. In order to ascertain the stoichiometry of high pressure superhydride phase observed at 80-100 GPa after laser heating, we have compared the ideal mixture of Ce–H_2_ solution with experimental volume per formula unit of CeH_3_, CeH_9_, and with theoretical EOS (see Fig. 6a). The curve representing ideal mixture of Ce and (7/2) H_2_ lies well below the theoretical and experimental EOS of *P6*_*3*_*/mmc*-CeH_9_ in the pressure range 80–100 GPa, whereas  mixture of Ce and (8/2) H_2_ partially overlaps with theoretical and experimental EOS of CeH_9_. This indicates that the hexagonal phase observed at 80-100 GPa after laser heating does not favour energetically CeH_*X*_ with *x* < 8 and can decompose into its elemental constituents or hydride with *x* > 8 and hydrogen. On the other hand, the curve representing an ideal mixture of Ce and (9/2) H_2_ lies well above the theoretical and experimental EOS of CeH_9_ in the pressure range 80–100 GPa. This observation clearly indicated that CeH_9_ can be stabilized in the pressure range mentioned. From our theoretical calculations and energetic considerations, it clearly signifies that the hexagonal phase observed at 80-100 GPa with laser heating has the CeH_9_ stoichiometry. We can also see that there is a fair agreement between experimental volume and theoretical EOS results (Fig. 6a) for $P6_3/mmc\mbox {-}{\mathrm{CeH}}_{\mathrm{9}}$ as well as $\beta \mbox {-}Pm\bar 3n\mbox {-}{\mathrm{CeH}}_{\mathrm{3}}$.

In hydrides, hydrogen is pre-compressed by interaction with metal atoms. Hydrogen sublattice in hydrides might be expected to be identical with atomic metallic hydrogen. So, we compared the volume expansion (Δ*V*_H_) per hydrogen atom and nearest H–H distance in CeH_9_ with simulated atomic metallic hydrogen phase extrapolated to lower pressure^31^ and also with other reported hydrides as shown in Fig. 6b, c, respectively^31,32,41,42^. The nearest Ce–H distance in $P6_3/mmc\mbox {-}{\mathrm{CeH}}_{\mathrm{9}}$ is plotted in Fig. 6d. The inset of Fig. 6d shows the Ce–H_29_ clathrate cage structure in which the H_29_ cage is encapsulating a cerium atom. Δ*V*_H_ per H atom for CeH_9_ is 2.09 Å^3^ at 100 GPa, which is lower than that for a hydrogen atom, but larger by ~8% with respect to Δ*V*_H_ per H atom of layered FeH_5_. Δ*V*_H_ per H atom for CeH_9_ matches with volume of atomic metallic hydrogen around 100 GPa. Formation of CeH_9_ can also be understood in terms of mixing of Ce and dense atomic metallic hydrogen. This indicates that hydrogen framework surrounding Ce atom is identical to dense atomic metallic hydrogen at a lower pressure. Nearest H–H distance in clathrate CeH_9_ is 1.116 Å at 100 GPa, which is significantly longer than the H–H bond length (0.74 Å) in H_2_ gas molecules but is significantly lower than other hydrides such as AlH_3_, FeH_3_, FeH_5_, H_3_S, and LaH_10_ as can be seen in Fig. 6c. Surprisingly, the nearest H–H distance in CeH_9_ almost overlaps with the H–H distance in atomic hydrogen and decreases very slowly with pressure. Among all the superhydrides, the nearest H–H distance observed in CeH_9_ is among the shortest  at 100 GPa and coincides with H–H distance of atomic metallic hydrogen. Among the reported hydrides, nearest H–H distance of CeH_9_ is only second to the H–H distance (0.98 Å)^23^ for atomic metallic hydrogen at 500 GPa at which hydrogen is in a superconducting metallic state^43^. Judging from the H–H distances, CeH_9_ is closer to monatomic metallic hydrogen than other hydrides, yet its predicted *T*_c_ is not as high as that of hydrogen or of LaH_10_. The shortest H-H distance is not the only thing that matters for high *T*_c_; the electronic structure of the metal atom plays a crucial role, as shown by Semenok et al.^29^. In Fig. 6c, we can also see that non-superconducting FeH_5_ has shorter H–H distance than H_3_S compound well known for very high *T*_c_. Presence of strongly coupled hydrogen-dominant libration and stretch vibrations are the signatures of high-*T*_c_ in hydrogen-rich materials^44^. Weak H–H interactions with preferred bond distances between 1.2 and 1.3 Å, the stretching and bending vibrations becomes indistinguishable, due to which all H vibrations contribute in the strong electron–phonon coupling process, eventually contributing to enhance the *T*_c_ in hydrides^44^. At 100 GPa the nearest Ce–H distance is ~2.07 Å and it decreases with pressure. It is noteworthy that the clathrate structures predicted in the literature for rare earth (RE) hydrides REH_6_, REH_9_, and REH_10_ have H_24_, H_29,_ and H_30_ cages surrounding the RE atom^23^. Among these cages, The H_29_ cage has the smallest volume per formula unit for YH_9_ (ref. ^23^). Clathrate H_29_ cage in CeH_9_ surrounding the Ce atom is almost 1.1 Å thick along the *a*- and *b*-axis, while it is 0.9 Å thick along *c*- axis at 100 GPa^45^, whereas thickness of clathrate cage in LaH_10_ is 0.9 Å^32^. Clathrate CeH_9_ can be visualized as three-dimensional atomic metallic hydrogen encapsulating Ce atoms (inset of Fig. 6d). Covalently bonded hydrogen sublattice in CeH_9_ with bond length and Δ*V*_H_ per H atom similar to atomic metallic hydrogen is likely to have density similar to that for atomic hydrogen slab at 100 GPa. Hence $P6_3/mmc\mbox {-}{\mathrm{CeH}}_{\mathrm{9}}$ will be a good platform to investigate H–H properties to understand atomic metallic hydrogen. Recently, Carbotte et al. proposed a new technique to investigate superconductivity in high pressure hydrides and hydrogen based on optical properties, without four probes^46^. Superconductivity in $P6_3/mmc\mbox {-}{\mathrm{CeH}}_{\mathrm{9}}$ can also be evaluated using this optical technique.

**Fig. 7:**
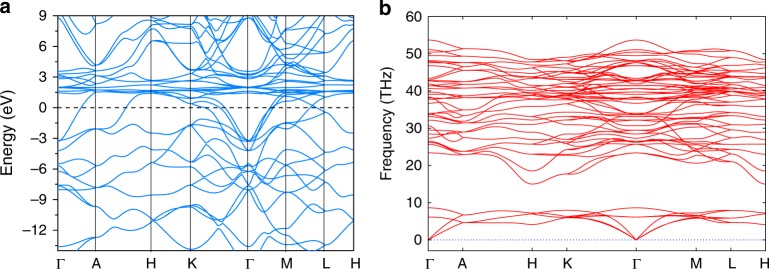
Electronic band structure and phonon dispersion curves for *P6*_*3*_*/mmc*-CeH_9_ at 150 GPa. **a** Electronic band structure for *P6*_*3*_*/mmc*-CeH_9_ at 150 GPa. Dotted line indicates Fermi level. **b** Phonon dispersion curves for *P6*_*3*_*/mmc*-CeH_9_ at 150 GPa. Absence of imaginary phonons in the dispersion curves shows the dynamical stability of *P6*_*3*_*/mmc*-CeH_9_ at 150 GPa. Phonon instability at 120 GPa is shown in Supplementary Fig. 8

**Fig. 8:**
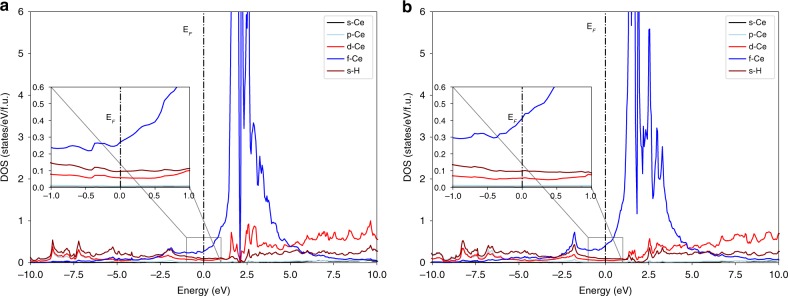
Electron density of states (DOS) for CeH_9_ at 150 GPa. **a** and **b** represents electron DOS computed with and without Hubbard correction respectively. Electron DOS at the Fermi level is largely dominated by Ce-4*f* and H orbitals

Figure 7a shows calculated electronic band structures of CeH_9_ at 150  GPa. From Fig. 7a, it can be seen that CeH_9_ is metallic and features numerous flat bands above the Fermi level. Noticeable density of electronic states at the Fermi level 0.73 (0.62) states per eV per f.u., which is 1.4 (1.2) times higher than that of previously found H_3_S^14^ and comparable to that of recently synthesized LaH_10_^32^ at an optimal pressure of 200 GPa, is a good sign for facilitating high-temperature superconductivity (values in parentheses are the corrected ones using GGA+U with U = 6 eV, see supplementary Table 2). Figure 8a, b represents the electron density of states (DOS) for CeH_9_ at 150 GPa with and without Hubbard correction respectively. The main contributors to *N*(*E*_f_) are Ce-4 and H-1*s* orbitals, however, only those electrons that are coupled strongly to phonons are important. High frequency phonons are mainly related to H vibrations, owing to its light mass, which makes the largest contribution to the electron–phonon coupling constant. Analysis of electron localization function (ELF) shows a moderate ELF value 0.64 between H atoms within the unit, suggesting weak covalent interaction, which forms a three-dimensional hydrogen network i.e., H_29_ cage consisting of H_4_, H_5_ and H_6_ rings. Very low ELF value between Ce and H indicates that no bonds were present between the Ce and H atoms (Supplementary Fig. 7).

We performed phonon calculations in the thermodynamic stability range of CeH_9_ i.e., above 80 GPa. Lattice dynamics calculations indicate that the *P6*_*3*_*/mmc*-CeH_9_ phase is dynamically stable at 150 GPa (Fig. 7b). Selected vibrational mode displacements of the *P6*_*3*_*/mmc*-CeH_9_ at the H- and K-point from the structure relaxed at 150 GPa are shown in the Supplementary Fig. 8. However, at lower pressures, e.g., 120 GPa and below, DFT calculations show that some phonon modes become imaginary along the **H**- and **K**-points ([−1/3, 2/3, 1/2] and [−1/3, 2/3, 0], respectively) (Supplementary Fig. 8). The *P6*_*3*_*/mmc*-CeH_9_ phase is stabilized anharmonically. To include temperature effect, capturing anharmonicity, we used the approach of Hellman et al.^47,48^, to obtain temperature-dependent phonon frequencies and the phonon spectra show dynamical stability already at 100 GPa and 500 K (see Supplementary Fig. 9). To resolve the soft modes of the lattice within the harmonic approximation, we used generalized evolutionary metadynamics (GEM)^49^, in which large displacements along the softest mode eigenvectors are used to equilibrate the system. This hybrid technique is implemented in USPEX and was successfully applied to boron and silicon and found numerous energetically competitive configurations^49^. We used supercells up to index 4, i.e., 80 atoms per cell. Using GEM, we found a stable structure within harmonic approximation with *C2/c* symmetry (Supplementary Fig. 10), which is a subgroup of *P6*_*3*_*/mmc* symmetry. Electron–phonon coupling (EPC) calculations revealed that *P6*_*3*_*/mmc*-CeH_9_ is a high-temperature superconductor. Using the Allen-Dynes modified McMillan equation (Eq. 1), we estimated the superconducting transition temperature (*T*_c_) to be 105–117 K at 200 GPa, when using different Coulomb pseudopotentials (*μ∗*), i.e., 0.10 and 0.15 which are widely accepted lower and upper bound values. In *P6*_*3*_*/mmc*, the resulting electron–phonon coupling coefficient *λ* is 2.30 at 200 GPa, which is higher than that of H_3_S, *λ* = 2.19 at 200 GPa^14^. Since cerium atom is heavy, the logarithmic average phonon frequency (*ω*_log_ = 740 K) is lower compared with that of H_3_S (*ω*_log_ = 1335 K), which results in a lower *T*_c_ of 105–117 K. The *T*_c_ of CeH_9_ has a lower value of 63–75 K at 100 GPa for *C2/c* structure. The logarithmic average phonon frequency *ω*_log_ of the *C2/c* phase has a lower value 662 K. However, our results indicate that lower *T*_c_ value is mainly related to the lower electron–phonon coupling coefficient *λ* = 1.48. Earlier report by Peng et al.^23^ predicted a slightly lower *T*_c_ value of 56 K at 100 GPa for the CeH_9_ phase with *P6*_*3*_*/mmc* structure^23^. However, our phonon calculations within harmonic approximation indicate instability of *P6*_*3*_*/mmc* phase below 120 GPa (see Supplementary Fig. 8), and it is unclear how *T*_c_ of a dynamically unstable phase was computed. So, our systematically carried studies estimate comparatively higher *T*_c_ of 63–75 K for the CeH_9_ at 100 GPa for the dynamically stable *C2/c* structure. Furthermore, we have tabulated the *ω*_log_, *λ*, and *T*_c_ value of CeH_9_ along with recently predicted other superhydrides of La-H, Y-H, U-H, Ac-H, and Th-H system for comparison, as shown in Supplementary Table 3. Phonon dispersions curves, phonon density of states, the Eliashberg spectral function *α*^*2*^*F(ω)*, and the EPC parameter *λ* as a function of frequency are calculated and shown in Supplementary Figs. 11 and 12 for *C2/c* and *P6*_*3*_*/mmc*-CeH_9_ at 100 and 200 GPa, respectively. It is known that quantum effects can impact the calculated superconducting transition temperatures; however, in the case of strongly anharmonic H_3_S (SG $Im\bar 3m$), the inclusion of anharmonic correction, lowered the *T*_c_ from its harmonic 204 K value^14^ only to 194 K^50^ at 200 GPa, and both are close to the reported experiment *T*_c_ at 200 GPa^15^, although the transition pressures shift is considerably large^14,51^.

In summary, we have successfully synthesized a cerium superhydride phase of CeH_9_ at 80-100 GPa after laser heating, which crystallized in the hexagonal *P6*_*3*_*/mmc* clathrate structure. In addition to this we have also synthesized a cubic phase of CeH_3_ with space group $Pm\bar 3n$ (*β*-UH_3_ type), which was recovered at ambient phase after complete decompression. Our studies give strong evidence for the synthesis of rare earth superhydrides and pave the way for future studies on other rare earth-hydrogen systems under extreme pressure with the aid of laser heating perhaps to make the binary hydrides *M*H_*x*_ with *x* > 9. Apart from this, the estimated *T*_c_ of 105–117 K in *P6*_*3*_*/mmc*-CeH_9_ at 200 GPa is very promising. Electron–phonon coupling in *P6*_*3*_*/mmc*-CeH_9_ is even higher than in H_3_S but still could not achieve higher *T*_*c*_ due to lower logarithmic average phonon frequency. Conspicuously, the dense three-dimensional atomic hydrogen sublattice is noted for superhydride *P6*_*3*_*/mmc*-CeH_9_ as compared with reported super/polyhydrides and similar to atomic metallic hydrogen at 100 GPa. The discovery of CeH_9_ at a feasible pressure range with prediction of superconductivity will certainly inspire further studies on superconductivity in hydride systems.

## Methods

### Experimental details

High pressure–temperature (*P*–*T*) experiments were carried out using a single-sided laser-heated DAC with a pair of bevelled diamond anvils of size 100–300 µm culets. Polycrystalline cerium (*Alfa Aesar*, 99.9% purity) sample of ~5 µm thickness was loaded inside a sample chamber drilled to a diameter of 75 µm in a rhenium gasket of 250 µm initial thickness pre-indented to 18 µm. Cerium is very likely to oxidize in open air so the sample was loaded in an argon filled glove box where both H_2_O and O_2_ concentrations were maintained below 0.1 ppm. A small piece of gold (~5 µm width) was also placed near the sample for pressure calibration as shown in Supplementary Fig. 13. For hydrogen loading, sample chamber was initially sealed by slightly closing the gasket and then opened in the high-pressure gas loading system in order to fill it with high purity hydrogen gas at room temperature under ~1.7 kilobar pressure. After hydrogen loading, Raman spectra of the H_2_ vibron, collected at 9 GPa from the sample chamber, confirmed the presence of H_2_ inside the sample chamber (Supplementary Fig. 14). XRD patterns were recorded at beamline 13-IDD of GSECARS at the Advanced Photon Source. Angle-dispersive XRD patterns were recorded on a PILATUS CdTe 1M detector with a synchrotron radiation of incident wavelength 0.3344 Å focused to a spot size of ~3 × 4 µm (FWHM). A clean-up slit with an 8 µm size pin-hole was used to cut down the beam tails and collect the XRD from the smallest area possible. Pulsed-laser heating was carried out using the online infrared laser set-up with a wavelength of 1064 nm available at beamline 13-IDD^52^. Several cycles of laser heating were carried at each pressure of 33, 60, 80, 89, 98, and 100 GPa during compression and at 79 and 54 GPa during the decompression run. The use of the pulse laser heating not just helped to promote reaction between Ce and H_2_ to form cerium hydrides but also reduced the possibility of diamond anvil failure. Pulsed-laser heating with microsecond pulse width has been utilized to reach temperatures of 1000–2000 K. Every laser heating shot was formed by accumulating 300k frequency modulated laser pulses of one microsecond pulse width at a rate of 10 kHz. The flat top of the laser heating spot size was around 10 µm in diameter. We strictly avoided temperatures above 2000 K to protect diamonds as hydrogen loaded DACs at high *P*–*T* conditions are most likely to fail^35,53^. Maintaining the sample temperature below 2000 K and relatively cold surrounding area using pulsed heating mode helped to avoid contamination and parasitic reaction with the sample chamber wall of gasket. Also, the laser heating spot on the sample was consistently maintained at substantial distance from gasket wall to avoid any contamination due to unwanted reaction (see Supplementary Fig. 15). In situ temperature measurements were carried by fitting the slope of thermal radiation spectra to a Planck radiation function. Uncertainty in temperature measurements were less than ±100 K. Obtained raw images of XRD were integrated with DIOPTAS software^54^. Rietveld and Lebail refinements were carried out using FullPROF software.

### Computational  details

Evolutionary variable-composition simulation, implemented in USPEX, is used to explore the high-pressure phase diagram of the Ce–H system from ambient pressure to 250 GPa. The evolutionary algorithm USPEX^55–59^ is a powerful method for finding thermodynamically stable compounds of a given system and their most stable structures. This method has been shown to be successful in predicting high-pressure structures of variety of systems which were confirmed experimentally, also in a number of in specific superconducting hydrides, e.g., UH_8_^33^ and H_3_S^14^. In this method, the first generation of structures (100 structures) and compositions are created using the random symmetric algorithm. Subsequent generations were obtained using 40% heredity, 20% transmutation, 20% softmutation, and 20% random symmetric generator. We allowed variation operators to automatically evolve in the subsequent generations. The underlying structure relaxations were carried out using the VASP package^59^ in the framework of DFT and using PBE-GGA (Perdew–Burke–Ernzerhof generalized gradient approximation)^60,61^. We believe PBE is the most appropriate choice, because PBE best reproduces the experimental data (see Supplementary Fig. 16 and Supplementary Tables 4 and 5). The projector-augmented wave approach (PAW)^59,62^ was used to describe the core electrons and their effects on valence orbitals. Valence electron configuration of 5*s*^2^5*p*^6^4*f*^1^5*d*^1^6*s*^2^ (i.e., with explicitly included *f* electrons) and 1*s*^1^ was used for the Ce and H atoms, respectively. A plane-wave kinetic-energy cut-off of 1000 eV for hard PAW potentials and dense Monkhorst–Pack k-points grids with reciprocal space resolution of 2*π* × 0.03 Å^−1^ were employed^63^ to sample the Brillouin zone. Phonon frequencies and superconducting critical temperature were calculated using density-functional perturbation theory as implemented in the QUANTUM ESPRESSO package^64^, also using the PBE-GGA functional. Ultrasoft pseudopotentials for Ce and H were used with a plane-wave basis set cut-off of 70 Ry, which gives a convergence in energy with a precision of 1 meV per atom. Phonon dispersions were also calculated under the quasi-harmonic approximation using the finite displacement method implemented in PHONOPY package^65^ using forces computed with VASP. The *k*-space integration (electrons) was approximated by a summation over a 12 × 12 × 6 uniform grid in reciprocal space, with the smearing scheme of Methfessel–Paxton and a fictitious smearing temperature *T* of *k*_B_*T* = 0.05 Ry for self-consistent cycles and relaxations; a much finer (24 × 24 × 12) grid was used for evaluating DOS and electron–phonon linewidths. Dynamical matrices and electron–phonon linewidths of *P6*_*3*_*/mmc*-CeH_9_ were calculated on a uniform 6 × 6 × 3 grid in q-space. Electron–phonon matrix elements were calculated based on interpolation method developed by Wierzbowska et al.^66^. The superconducting transition temperature *T*_c_ was estimated using the Allen–Dynes modified McMillan equation^67^

1 $$T_{\mathrm{c}} = \frac{{\omega _{{\mathrm{{log}}}}}}{{1.2}}{\mathrm{{exp}}}\left( {\frac{{ - 1.04(1 + \lambda )}}{{\lambda - \mu ^ \ast (1 + 0.62\lambda )}}} \right),$$

where *μ*^*∗*^ is the Coulomb pseudopotential and *ω*_log_ is the logarithmic average phonon frequency. The electron–phonon coupling constant *λ* and *ω*_log_ were calculated as

2 $$\omega _{\mathrm{{log}}} = {\mathrm{exp}}\left( {\frac{2}{\lambda }{\int} {\frac{{{\mathrm{d}}\omega }}{\omega }\alpha ^2F\left( \omega \right){\mathrm{{ln}}}(\omega )} } \right),$$

3 $$\lambda = 2 {\int_0^{\infty}} {\frac{{{\alpha}^2 F\left({\omega} \right)}}{\omega}{\mathrm{d}}{\omega}}.$$

## Supplementary information

Supplementary Information

Peer Review File

## Data Availability

All data supporting the findings of this study are included in this article and its Supplementary Information, and are also available from the corresponding authors upon request. Data are deposited on https://figshare.com/ and can be found on the link—10.6084/m9.figshare.9729467.v3
