# Supplementary material for: Synthesis of clathrate cerium superhydride CeH9 at 80-100 GPa with atomic hydrogen sublattice
Source: Nat Commun. 2019 Oct 1;10:4453. doi: 10.1038/s41467-019-12326-y (PMC6773858; doi:10.1038/s41467-019-12326-y)
Supplement: Supplementary file 1 — Supplementary Information [file 41467_2019_12326_MOESM1_ESM.pdf]

Supplementary Information

Synthesis of clathrate cerium superhydride CeH_9 at 80-100 GPa with atomic hydrogen sublattice

Nilesh P. Salke *et al.*

Supplementary Note-1

XRD pattern of the sample initially loaded at 9 GPa could be indexed with $Fm\bar{3}m$ structure. From the literature, it is well known that at ambient P-T conditions CeH_X system can stabilize in various stoichiometries ranging from X = 2 to 3[1]. Specifically, two extreme compositions CeH_2 and CeH_3 stabilize in $Fm\bar{3}m$ structure whereas $CeH_{2.5}$ (Ce_2H_5) forms tetragonal structure with $I4_1md$ space group[1]. Besides this, CeO_2, which could occur due to starting sample being contaminated and oxidized, also has fluorite type $Fm\bar{3}m$ structure[2]. It was difficult to confirm the exact stoichiometry and composition of observed $Fm\bar{3}m$ phase at 9 GPa. It was also important to confirm stoichiometry and composition of the sample to rule out any contamination. Reported values of lattice parameter at ambient pressure for $Fm\bar{3}m$ structured CeH_2 and CeH_3 were 5.580 and 5.534 Å respectively[3]; corresponding volume per formula unit (f.u.) is 43.435 and 42.37 Å^3 respectively[3]. We fitted the experimental volume per f.u. (assuming Z=4) of observed $Fm\bar{3}m$ phase using third order Birch-Murnaghan EOS (see supplementary Figure 1d). Zero pressure volume obtained after fitting is V_0 = 44(1) Å^3 per f.u., which is closer to the reported V_0 per f.u. of CeH_2, which rules out the $Fm\bar{3}m$-CeH_3 phase. In supplementary Figure 1d, we also compared the experimental EOS with reported EOS of CeO_2[2]. From supplementary Figure 1d, it clearly indicates that experimental EOS of $Fm\bar{3}m$ phase is not consistent with EOS of $Fm\bar{3}m$-CeO_2 which firmly rules out the presence of $Fm\bar{3}m$-CeO_2 in the sample. Hence, we concluded that $Fm\bar{3}m$ structure observed in the beginning at 9 GPa was of CeH_2 phase which persisted up to 33 GPa until we carried laser heating on the sample.

Supplementary Note-2

In the second experimental run, we carried the elemental reaction between cerium powder and hydrogen inside a laser heated diamond anvil cell. We observed the phases at P-T conditions similar to our previous run. Supplementary Figure 6 shows the XRD patterns of various phases that we synthesized in our experiments. In the beginning, we observed the $Fm\bar{3}m$-CeH_2 phase at 32 GPa at ambient temperature. Further with compression at 51 GPa and with laser heating of ~2000 K, we synthesised the β-UH_3 type (space group $Pm\bar{3}n$) CeH_3 Phase (β-$Pm\bar{3}n$-CeH_3). Finally, above 82 GPa with pulse laser heating of ~2500 K, we observed a Cerium superhydride CeH_9 phase with hexagonal $P6_3/mmc$ structure. Unfortunately, the diamonds broke at 84 GPa during further compression.

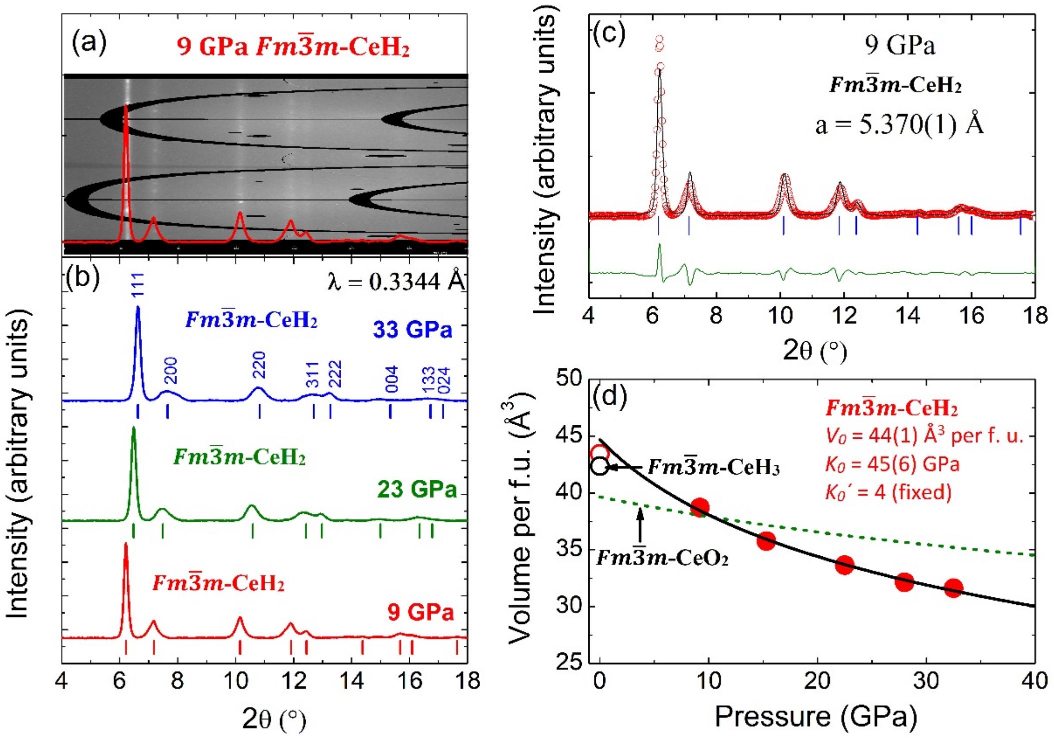

Supplementary Figure 1: Representative x-ray diffraction patterns at high pressures and equation of state of $Fm\bar{3}m$-CeH_2. (a) XRD image at 9 GPa representing polycrystalline nature of $Fm\bar{3}m$-CeH_2 phase. (b) X-ray diffraction patterns of $Fm\bar{3}m$-CeH_2 from 9 to 33 GPa. Red, green and blue vertical ticks correspond to peak positions of $Fm\bar{3}m$-CeH_2 phase at 9, 23 and 33 GPa respectively. (c) Lebail refinement plot for $Fm\bar{3}m$-CeH_2 at 9 GPa, $\chi^2 = 5.41$ (d) Volume per formula unit of $Fm\bar{3}m$-CeH_2 as a function of pressure is fitted using third-order Birch-Murnaghan equation of state (EOS). Red solid circles represent experimental unit cell volume per formula unit data. Error in volume is smaller than circle size. Black line is EOS fit to experimental data. Red and black open circle represents reported volume per formula unit of $Fm\bar{3}m$ structured CeH_2 and CeH_3 at ambient pressure respectively.[3] Green dotted line represents reported EOS for $Fm\bar{3}m$-CeO_2.[2]

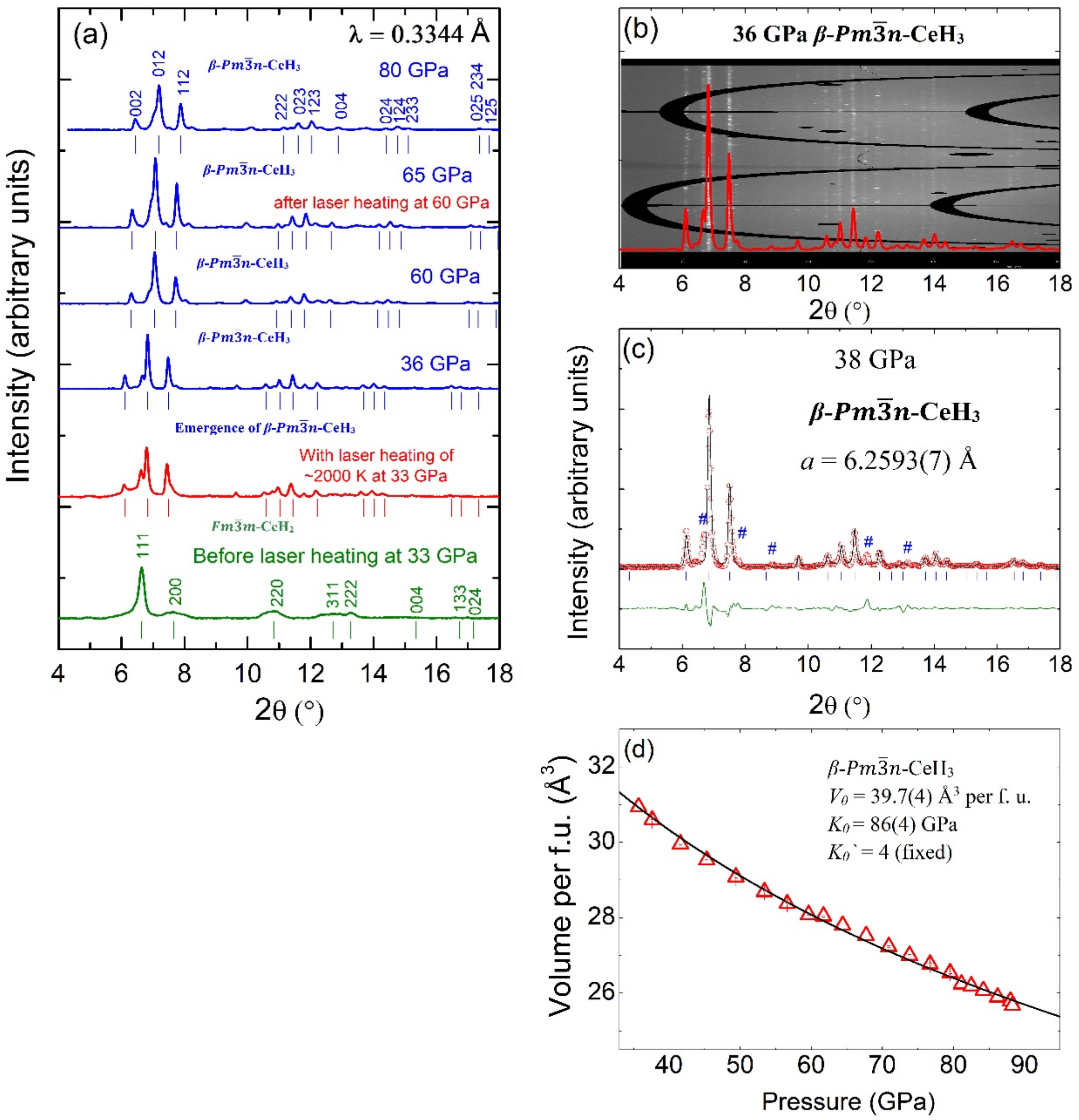

Supplementary Figure 2: Representative x-ray diffraction patterns denoting evolution of *β*-*Pm*$\bar{3}$*n*-CeH_3 under pressure with laser heating and equation of state fitting. (a) X-ray diffraction patterns at various pressures representing the formation of *β*-*Pm*$\bar{3}$*n*-CeH_3 up to 80 GPa. *β*-*Pm*$\bar{3}$*n*-CeH_3 phase forms after laser heating at 33 GPa and 2100 K. Vertical green ticks indicate indexing of peak position for *Fm*$\bar{3}$*m*-CeH_2; vertical red and blue ticks indicate indexing of peak position for *β*-*Pm*$\bar{3}$*n*-CeH_3 at respective pressure. (b) XRD image at 36 GPa representing polycrystalline nature of *β*-*Pm*$\bar{3}$*n*-CeH_3. (c) Lebail refinement plot for *β*-*Pm*$\bar{3}$*n*-CeH_3 at 38 GPa, χ^2 = 10.6, blue hash symbol represents unidentified peaks (d) Third-order Birch-Murnaghan equation of state fitting for *β*-*Pm*$\bar{3}$*n*-CeH_3. Red open triangles represent experimental data for *β*-*Pm*$\bar{3}$*n*-CeH_3, black solid line represent EOS fit. Fitting errors are also plotted in (d) but are mostly too small to be seen.

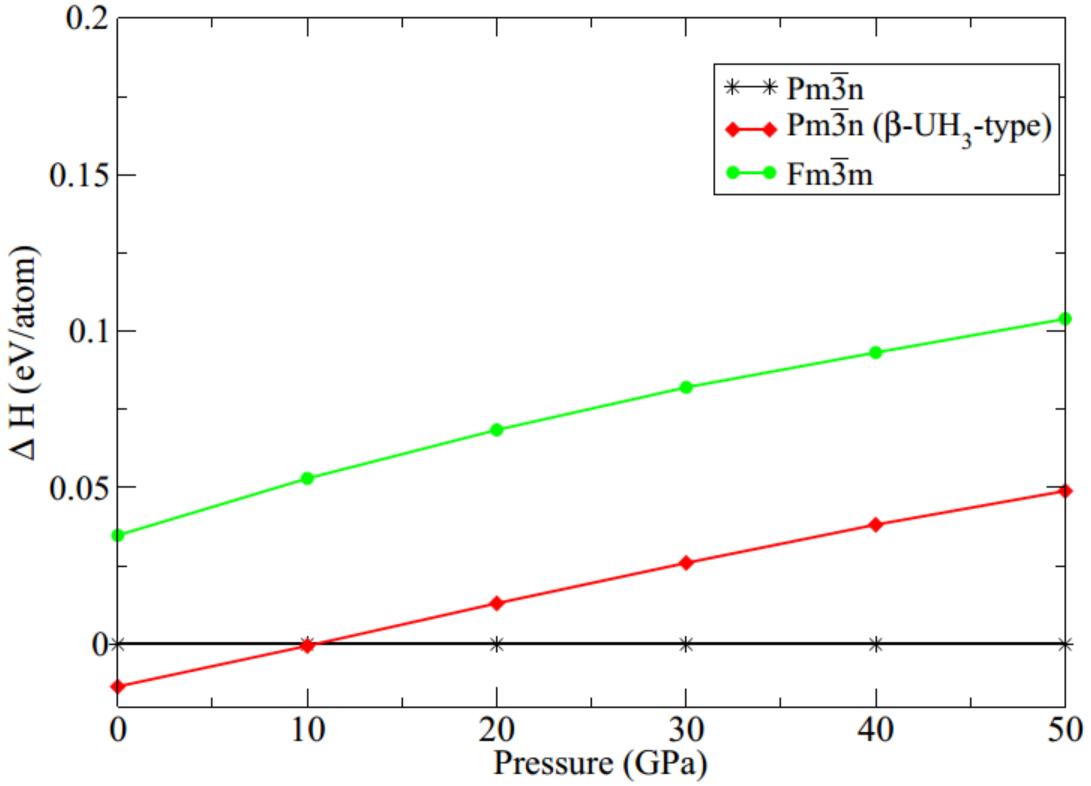

Supplementary Figure 3: Enthalpy comparison for various CeH_3 phases as a function of pressure. Enthalpy per atom relative to the $Pm\bar{3}n$ structure as a function of pressure for the best phases with the CeH_3 stoichiometry. Experimentally known ambient pressure phase $Fm\bar{3}m$-CeH_3 is added for comparison. Red, black and green symbols-line represents β-$Pm\bar{3}n$, $Pm\bar{3}n$ and $Fm\bar{3}m$ phases of CeH_3 respectively.

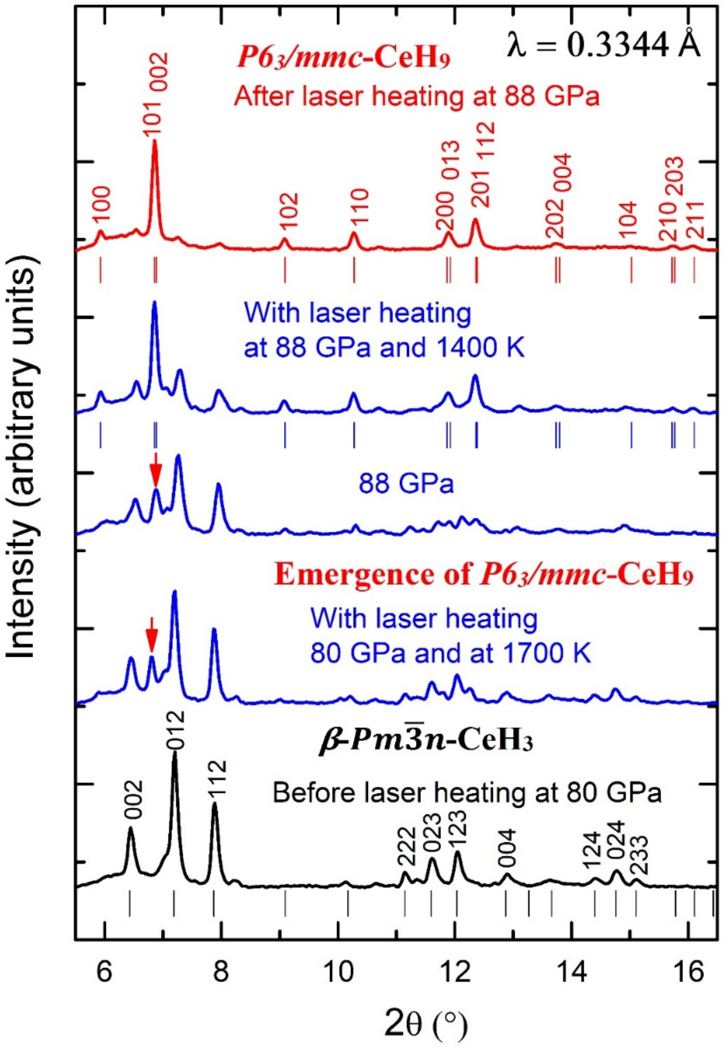

Supplementary Figure 4: X-ray diffraction patterns representing formation of superhydride *P6₃/mmc*-CeH₉. X-ray diffraction patterns at pressures above 80 GPa representing the formation of CeH_9 with laser heating. New peaks, which emerged after laser heating to 1700 K at 80 GPa, are shown with a red arrow corresponding to (101) and (002) of the *$P6_3/mmc$* phase. Vertical black ticks indicate indexing for β-$Pm\overline{3}n$-CeH_3 at 80 GPa. Vertical blue and red ticks indicate indexing of the *$P6_3/mmc$*-CeH_9 phase at the respective pressures.

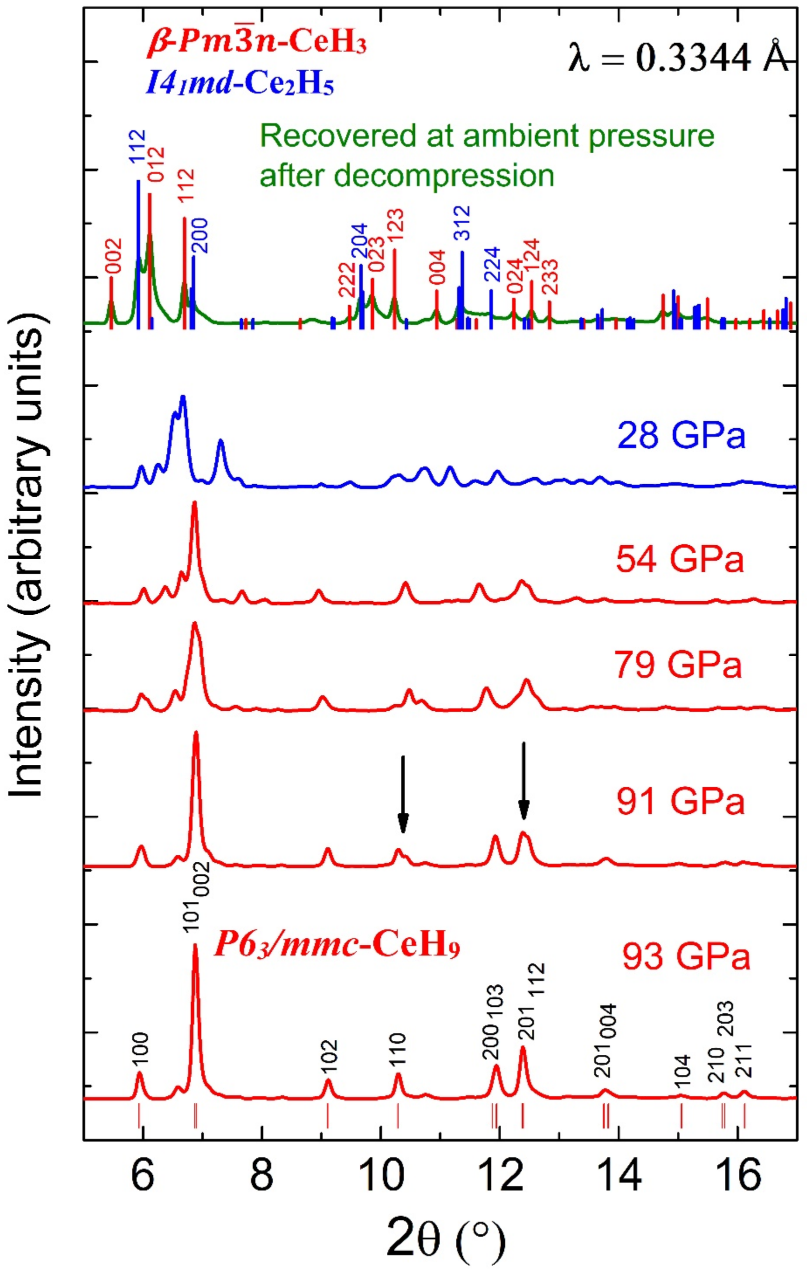

Supplementary Figure 5: Representative x-ray diffraction patterns obtained during the decompression cycle. XRD patterns were recorded upon the decompression cycle down to ambient pressure. Ambient pressure diffraction patterns of the recovered sample were recorded from the sample chamber after complete decompression. β-$Pm\overline{3}n$-CeH_3 and $I4_1md$-Ce_2H_5 phases were recovered at ambient conditions. Red vertical ticks for XRD at 93 GPa represent peak positions for $P6_3/mmc$-CeH_9. Black arrows for XRD at 91 GPa (ambient temperature) indicates the instability. Red and blue vertical lines for XRD of the recovered sample at ambient pressure indicate peak positions with relative intensity for β-$Pm\overline{3}n$-CeH_3 and $I4_1md$-Ce_2H_5, respectively.

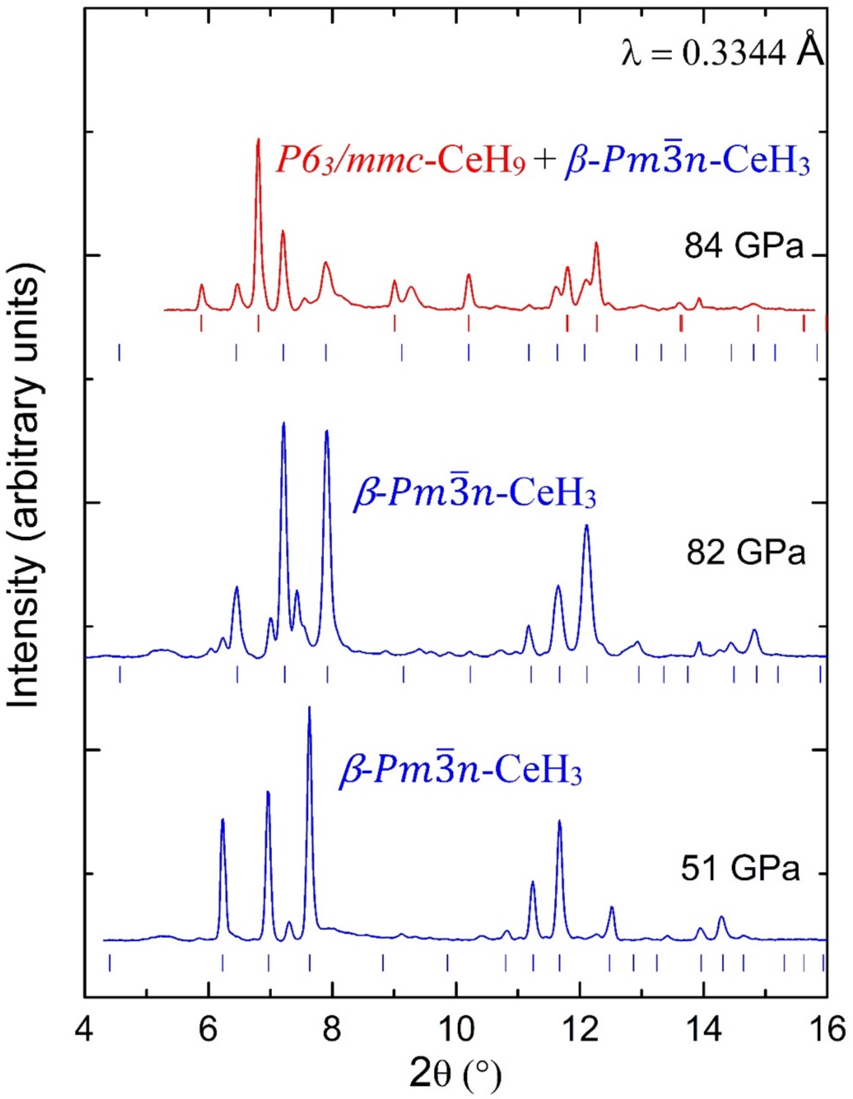

Supplementary Figure 6: Representative XRD patterns of various Cerium hydride phases in second experimental run. Blue and red vertical line indicates the indexing for β-$Pm\bar{3}n$-CeH_3 and $P6_3/mmc$-CeH_9 phases respectively.

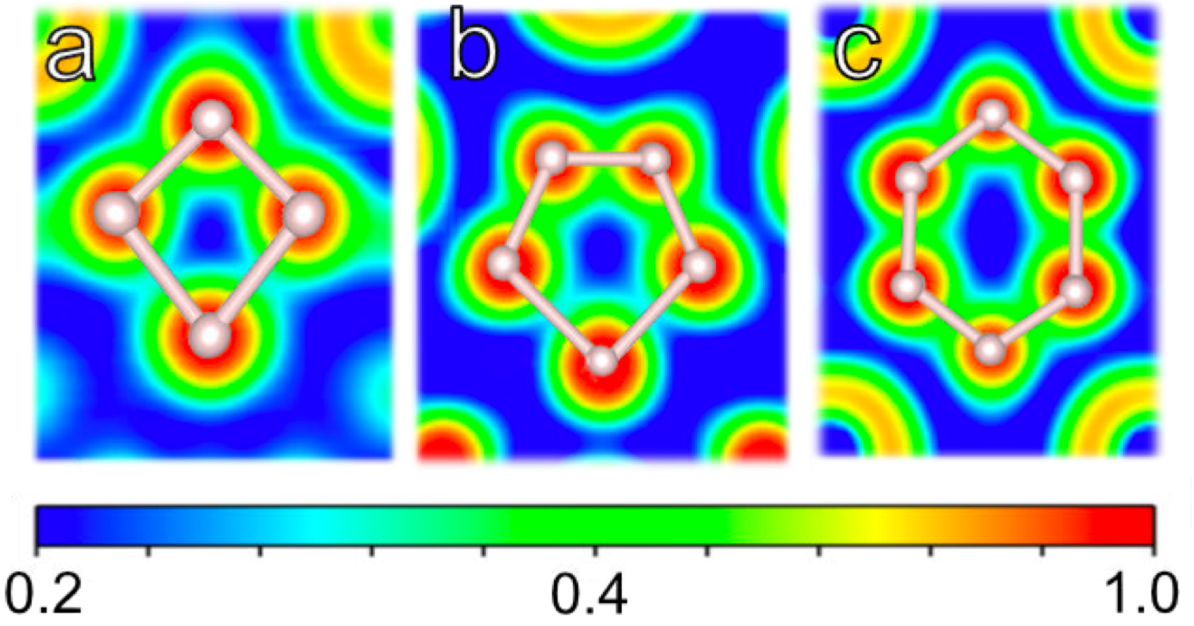

Supplementary Figure 7: Electron localization function (ELF) of CeH_9 at 150 GPa. Figure (a), (b) and (c) represents ELF plots in (1.15 0 1), (1 1 0) and (-1 0 2.55) sections for CeH_9 at 150 GPa. Hydrogen atoms and H_4, H_5 and H_6 rings are shown in the figures. For better clarity, we set the minimum of the ELF to 0.2 in the plots.

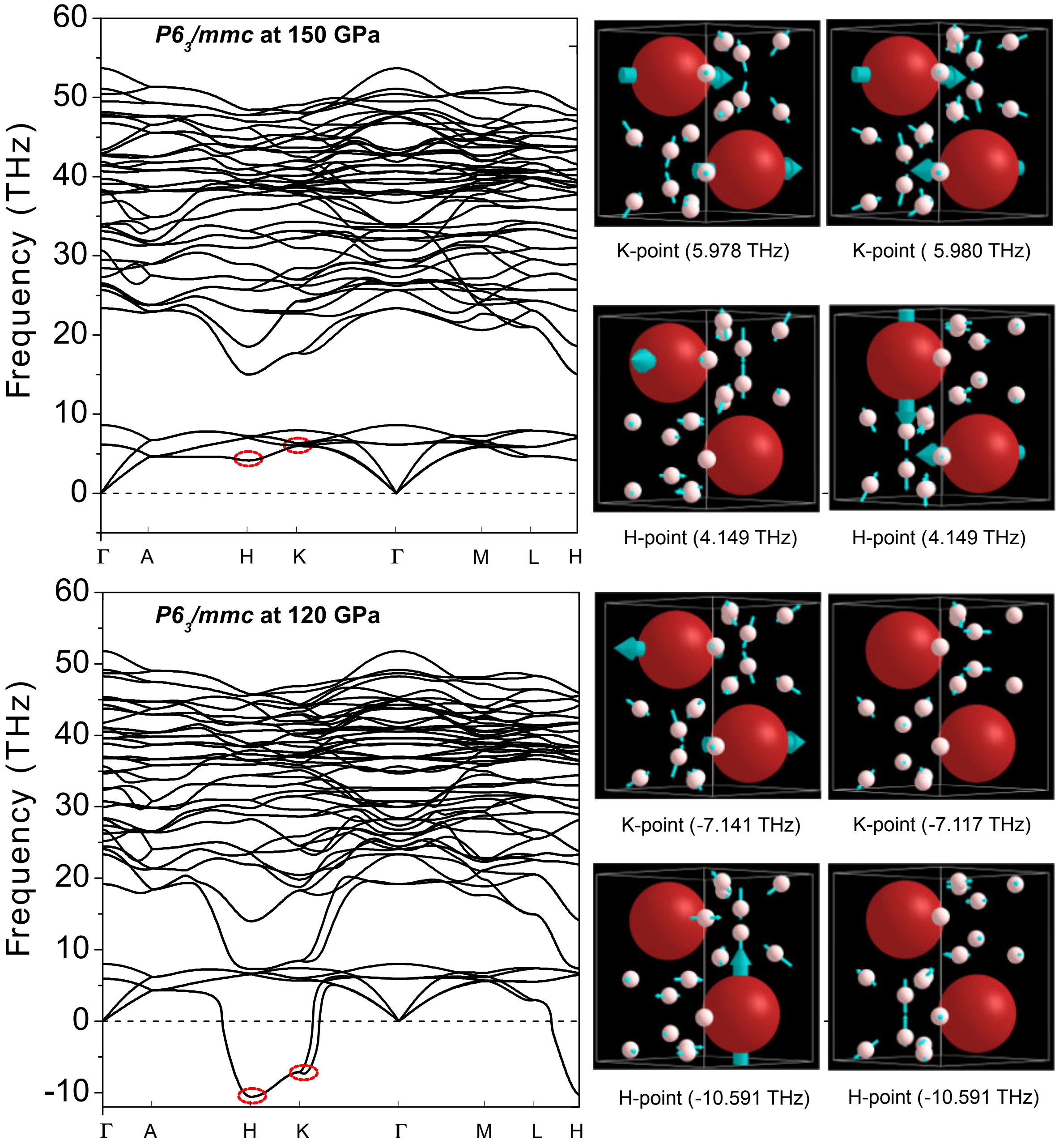

Supplementary Figure 8: Phonon dispersion curves and selected mode displacements of the *P6₃/mmc*-CeH₉ at 120 and 150 GPa. Unstable modes i.e., H- and K-point can be seen at 120 GPa. Large and small spheres show Ce and H atoms, respectively. Arrows refer to the displacement direction.

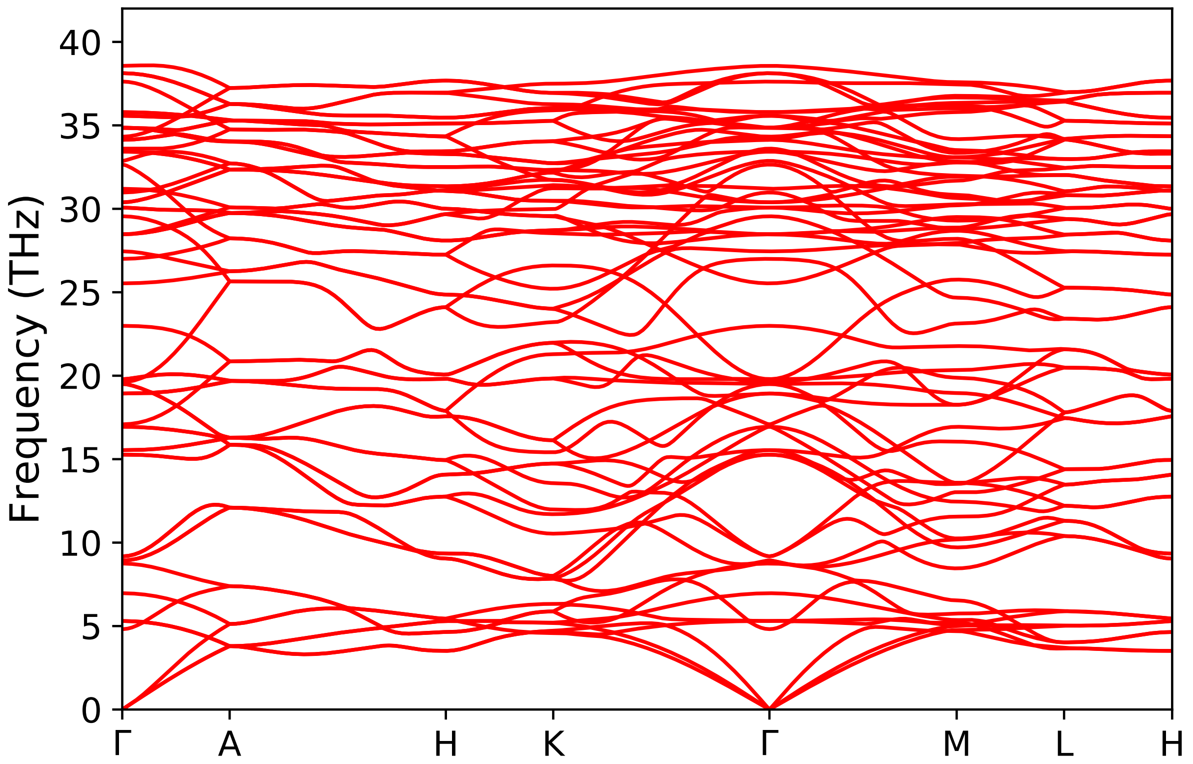

Supplementary Figure 9: Phonon dispersion curves for *P6₃/mmc*-CeH_9 at 100 GPa and 500 K. Absence of imaginary phonons in the dispersion curves shows the dynamical stability of *P6₃/mmc*-CeH_9 at 100 GPa and 500 K.

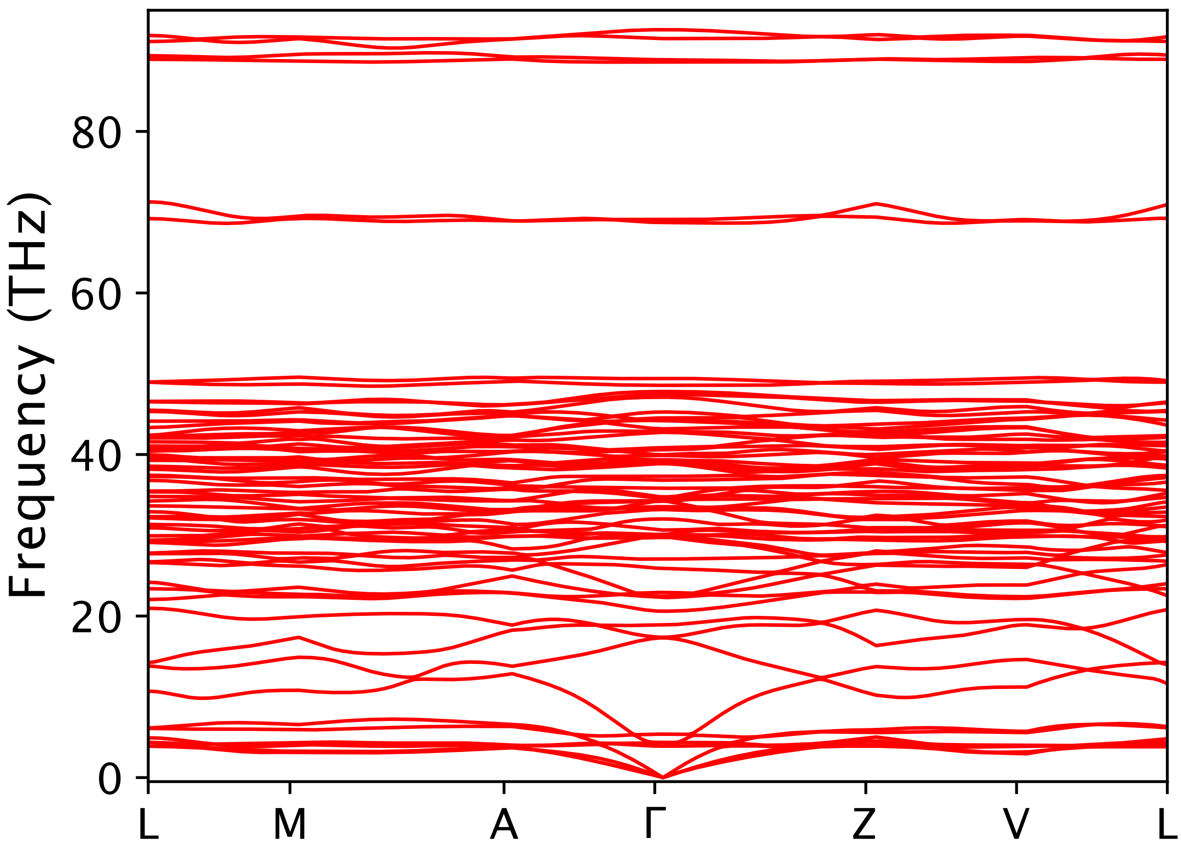

Supplementary Figure 10: Calculated phonon dispersion curves of *C2/c*-CeH_9 at 100 GPa. Phonon calculations show that lower symmetry structure *C2/c* does not have any imaginary frequency at 100 GPa, which simply refers to the stability of this phase. In addition to stability, lattice dynamics calculations show appearance of high frequency vibration of hydrogen atoms in the *C2/c* phase.

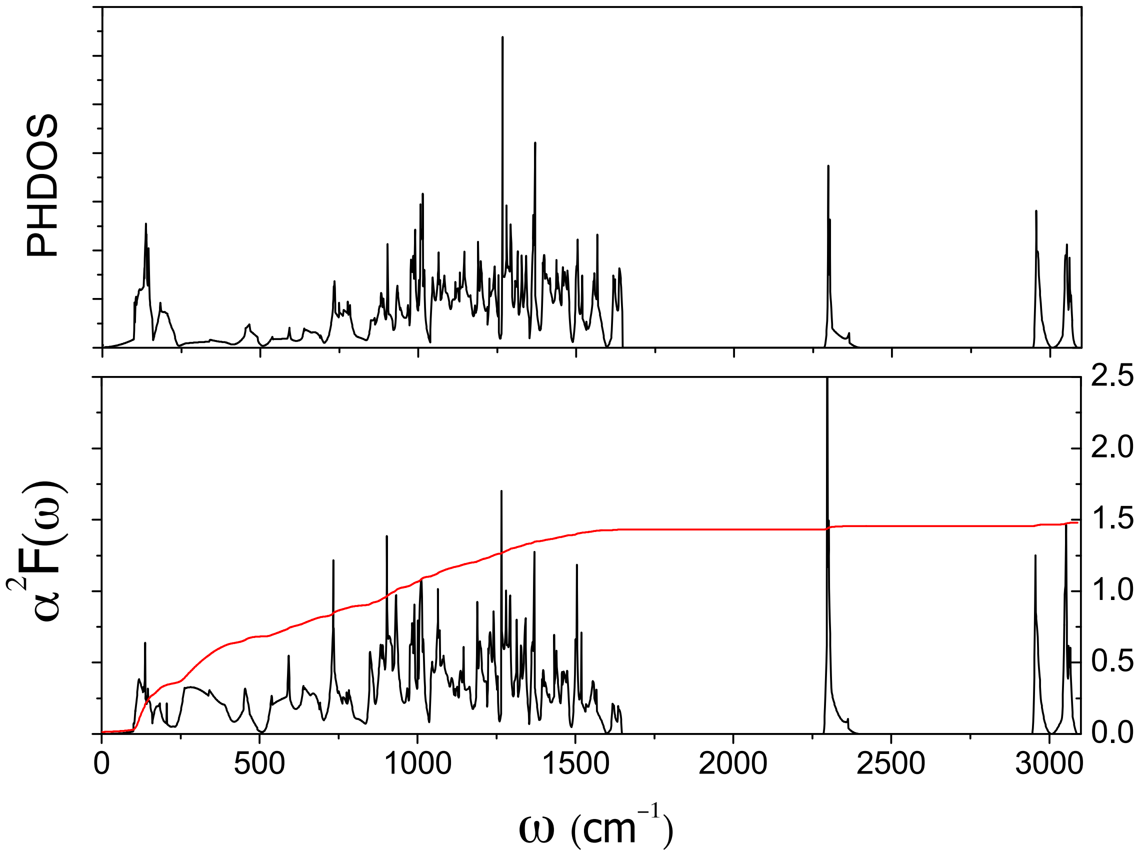

Supplementary Figure 11: (a) Calculated phonon density of states (PHDOS), (b) Eliashberg EPC spectral functions $\alpha^2F(\omega)$, and electron- phonon integral $\lambda(\omega)$ (red line) of *C2/c*-CeH_9 100 GPa. Both phonon density of states and Eliashberg spectral function show a gap between medium-frequency and high-frequency vibration of H atoms. The electron-phonon coupling coefficient (red line) shows that medium-frequency H modes contribute the most to the electron-phonon coupling.

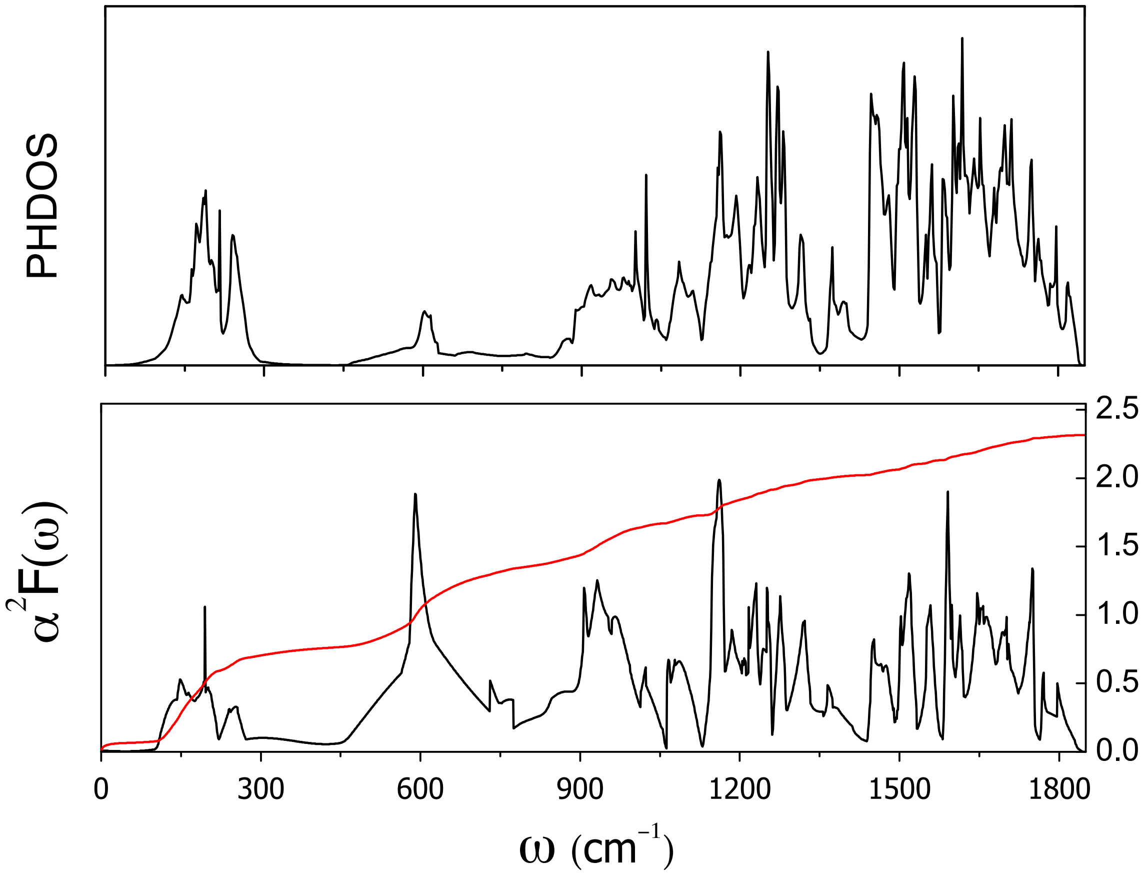

Supplementary Figure 12: (a) Calculated phonon density of states (PHDOS), (b) Eliashberg EPC spectral functions $\alpha^2F(\omega)$, and electron- phonon integral λ(ω) (red line) of *P6₃/mmc*-CeH₉ 200 GPa. Contrary to the low-symmetry phase of CeH_9, *P6₃/mmc* does not have hydrogen modes with frequency above 2000 cm^{-1}. The electron-phonon coupling coefficient (red line) shows that both medium-frequency and high-frequency H vibrations make a significant contribution to the electron-phonon coupling.

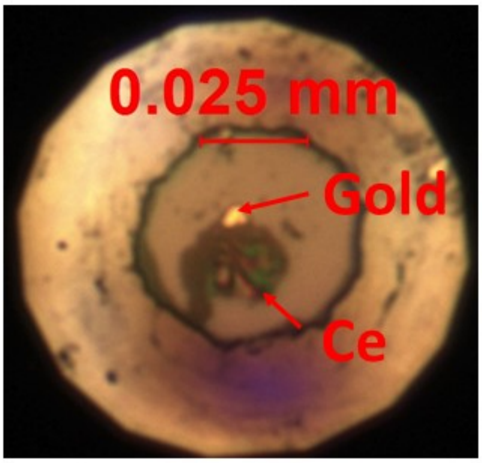

Supplementary Figure 13: Image of sample loading at 9 GPa. Image shows Ce (actually CeH_2) and Au surrounded by H_2 inside rhenium sample chamber at 9 GPa.

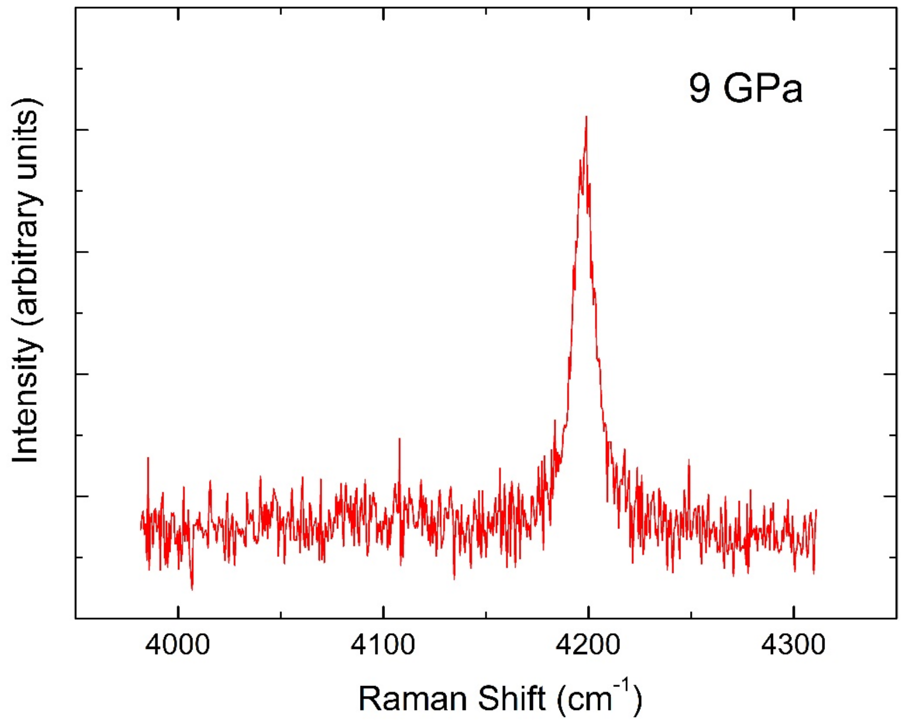

Supplementary Figure 14: Raman spectra recorded from sample chamber at 9 GPa. Raman spectra of H_2 vibron collected at 9 GPa from sample chamber confirming the presence of H_2 inside the sample chamber.

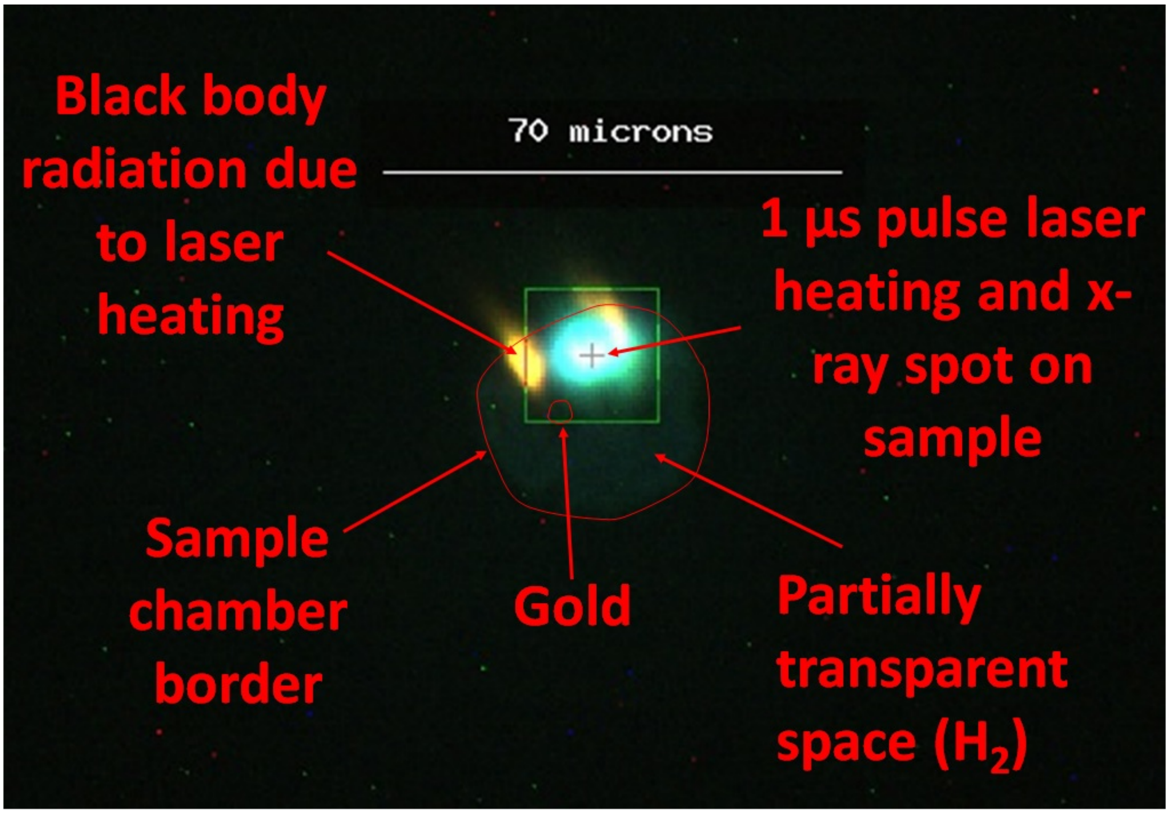

Supplementary Figure 15: Image of *in-situ* ~1 μs pulse laser heating and x-ray on sample at 100 GPa recorded at beamline 13-IDD of GSECARS at APS. Microsecond pulse laser heating of sample spot was always maintained at substantial distance from gasket corner (bright spot marked with an arrow) to avoid any unwanted reaction and contamination. Sample chamber edge and gold positions are marked with red line for clarity and labelled respectively in image. Partially transparent space shown with an arrow confirms the presence of hydrogen in sample chamber.

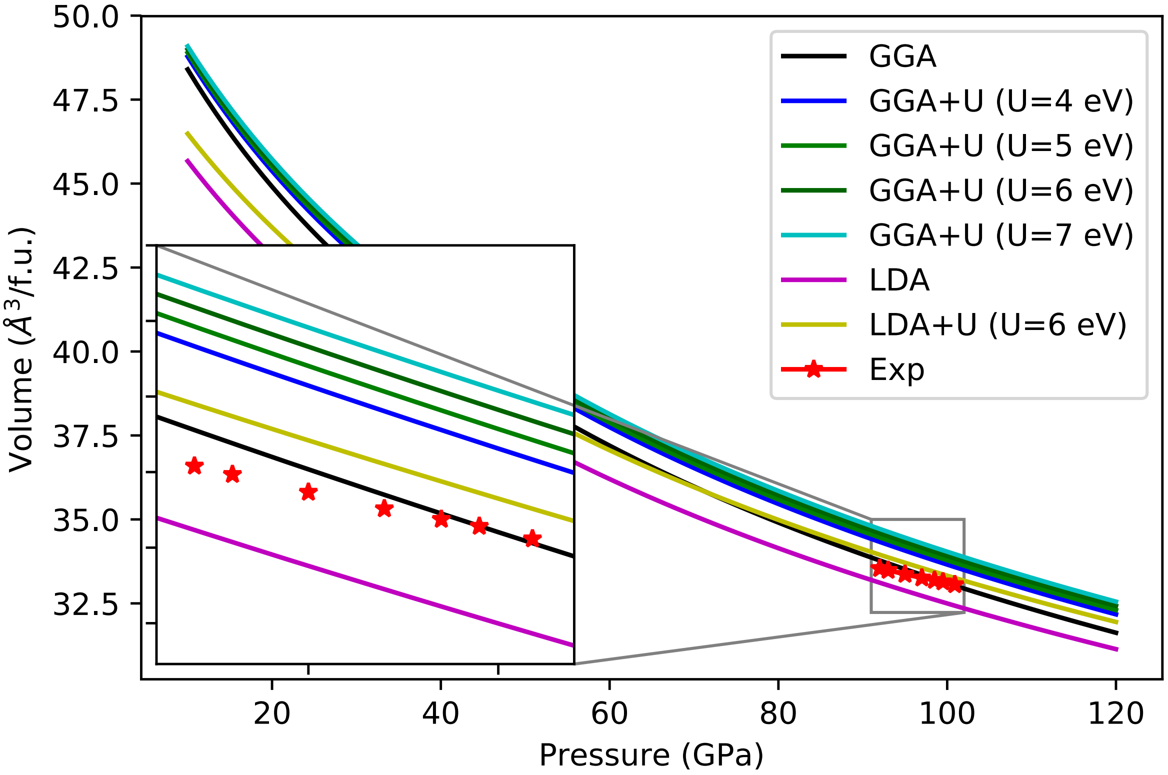

Supplementary Figure 16: Comparison of P-V data of various theoretical methods with experimental data.

Supplementary Table 1: Crystal structures parameters of the predicted Ce-H phases.

Phase	Space group	Lattice Parameters	Atoms	*x*	*y*	*z*
CeH_{10} (200 GPa)	$Fm\bar{3}m$	a = 4.877 Å	Ce (4a)	0.0000	0.0000	0.0000
			H (8c)	0.2500	0.2500	0.7500
			H (32f)	0.1227	0.1227	0.6227
CeH_9 (200 GPa)	$P6_3/mmc$	a = 3.497 Å	Ce (2d)	0.6667	0.3333	0.2500
		c = 5.224 Å	H (12k)	0.1550	0.8450	0.4351
			H (4f)	0.3333	0.6667	0.1464
			H (2b)	0.0000	0.0000	0.7500
CeH_9 (100 GPa)	$C2/c$	a = 3.685 Å	Ce (4e)	0.5000	0.0823	0.2500
		b = 6.427 Å	H (8f)	0.9242	0.8255	0.4396
		c = 6.652 Å	H (8f)	0.5502	0.8258	0.0613
		β= 123.60°	H (8f)	0.8168	0.0862	0.0656
			H (8f)	0.1152	0.0840	0.8596
			H (4e)	0.0000	0.2482	0.2500
CeH_8 (80 GPa)	$P6_3mc$	a = 3.727 Å	Ce (2b)	0.6667	0.3333	0.7743
		c = 5.576 Å	H (2b)	0.6667	0.3333	0.1520
			H (6c)	0.1746	0.3492	0.9512
			H (6c)	0.8434	0.6869	0.0910
			H (2a)	0.0000	0.0000	0.2745
CeH_6 (50 GPa)	$P6_3mc$	a = 3.704 Å	Ce (2b)	0.6667	0.3333	0.2894
		c = 5.733 Å	H (6c)	0.1778	0.3556	0.1077
			H (2a)	0.0000	0.0000	0.4456
			H (2a)	0.0000	0.0000	0.7915
			H (2b)	0.3333	0.6667	0.4093
CeH_4 (50 GPa)	$I4/mmm$	a = 3.044 Å	Ce (2b)	0.5000	0.5000	0.0000
		c = 6.021 Å	H (4e)	0.0000	0.0000	0.8657
			H (4d)	0.0000	0.5000	0.2500
CeH_3 (50 GPa)	$Pm\bar{3}n$	a = 3.815 Å	Ce (2a)	0.5000	0.5000	0.5000
			H (6d)	0.2500	0.5000	0.0000
CeH_3 (0 GPa)	$Pm\bar{3}n$ (β-UH_3-type)	a = 6.570 Å	Ce (2a)	0.0000	0.0000	0.0000
			Ce (6c)	0.2500	0. 0000	0.5000
			H (24k)	0.0000	0.1552	0.6953

Supplementary Table 2: Comparison of electronic density of states of various hydrides. We included U = 6 eV (GGA+U) and the corrected DOS at the Fermi level are reported in the parenthesis.

Hydride	Pressure (GPa)	*N(Ef)* (states per eV per f.u.)
$P6_3/mmc$-CeH_9	100 GPa	0.92 (0.73)
$P6_3/mmc$-CeH_9	150 GPa	0.81 (0.67)
$P6_3/mmc$-CeH_9	200 GPa	0.73 (0.62)
$Im\bar{3}m$-H_3S	200 GPa	0.52
$Fm\bar{3}m$ -LaH_{10}	200 GPa	0.73

Supplementary Table 3: Comparison of electron-phonon coupling coefficient (λ), logarithmic average phonon frequency (ω_{log}) and T_c for various hydrides.

Hydride	Space group	λ	ω_{log} (K)	T_c (K)	Pressure (GPa)	reference
CeH_9	$P6_3/mmc$	2.30	740	117 (μ^* = 0.10)<br>105 (μ^* = 0.15)	200	This study
CeH_9	$C2/c$	1.48	662	75 (μ^* = 0.10)<br>63 (μ^* = 0.15)	100	This study
H_3S	$Im\bar{3}m$	2.19	1335	203	200	4
YH_{10}	$Im\bar{3}m$	2.58	1282	326	250	5
LaH_8	$C2/m$	1.12	1591	131	300	5
LaH_{10}	$Fm\bar{3}m$	3.41	848	238	210	5
UH_7	$P6_3/mmc$	0.83	873.8	47.6	20	6
		0.95	764.9	57.5	0	
UH_8	$Fm\bar{3}m$	0.73	873.7	27.5	50	6
		1.13	450.3	37.6	0	
UH_9	$P6_3/mmc$	0.67	933.4	35.8	300	6
AcH_{10}	$R\bar{3}m$	3.46	710.9	204.1	200	7
AcH_{16}	$P\bar{6}m2$	2.16	1054	199.2	150	7
ThH_{10}	$Fm\bar{3}m$	2.19	1042.8	194.4	100	8

Supplementary Table 4: Comparison of lattice parameters and volume of *$P6_3/mmc$*-CeH_9 at 100 GPa by various DFT functionals

**Functional**	***a* (Å)**	***c* (Å)**	**Vol ($Å^3$ per f.u.)**
LDA	3.6827	5.5356	32.510
LDA+U (6 eV)	3.7072	5.6018	33.335
GGA	3.7022	5.5813	33.125
GGA+U (6 eV)	3.7259	5.6445	33.930
Experiment	3.7110	5.5429	33.053

Supplementary Table 5: Comparison of bulk modulus of *$P6_3/mmc$*-CeH_9 by various DFT functionals

**Functional**	**Bulk modulus K_0 (GPa)**	**V_0 (A^3 per f.u.)**	**K_0'**
LDA	98.1	49.9	4.0
LDA+U (6 eV)	105.4	50.4	4.0
GGA	80.5	53.4	4.0
GGA+U (6 eV)	84.4	54.0	4.0

Supplementary References

1 Avisar, D. & Livneh, T. Raman scattering by phonons and crystal-field excitations in cerium hydrides. *Journal of Alloys and Compounds* **494**, 11-16, doi:https://doi.org/10.1016/j.jallcom.2009.11.108 (2010).

2 Jacobsen, M. K., Velisavljevic, N., Dattelbaum, D. M., Chellappa, R. S. & Park, C. High pressure and temperature equation of state and spectroscopic study of CeO 2. *Journal of Physics: Condensed Matter* **28**, 155401 (2016).

3 Korst, W. L. & Warf, J. C. Rare Earth-Hydrogen Systems. I. Structural and Thermodynamic Properties. *Inorganic Chemistry* **5**, 1719-1726, doi:10.1021/ic50044a018 (1966).

4 Duan, D. *et al.* Pressure-induced metallization of dense (H2S)2H2 with high-Tc superconductivity. *Scientific Reports* **4**, 6968, doi:10.1038/srep06968 (2014).

5 Liu, H., Naumov, I. I., Hoffmann, R., Ashcroft, N. W. & Hemley, R. J. Potential high Tc superconducting lanthanum and yttrium hydrides at high pressure. *Proceedings of the National Academy of Sciences* **114**, 6990-6995, doi:10.1073/pnas.1704505114 (2017).

6 Kruglov, I. A. *et al.* Uranium polyhydrides at moderate pressures: Prediction, synthesis, and expected superconductivity. *Science Advances* **4**, eaat9776, doi:10.1126/sciadv.aat9776 (2018).

7 Semenok, D. V., Kvashnin, A. G., Kruglov, I. A. & Oganov, A. R. Actinium Hydrides AcH10, AcH12, and AcH16 as High-Temperature Conventional Superconductors. *The Journal of Physical Chemistry Letters*, 1920-1926, doi:10.1021/acs.jpclett.8b00615 (2018).

8 Kvashnin, A. G., Semenok, D. V., Kruglov, I. A., Wrona, I. A. & Oganov, A. R. High-Temperature Superconductivity in a Th–H System under Pressure Conditions. *ACS Applied Materials & Interfaces* **10**, 43809-43816, doi:10.1021/acsami.8b17100 (2018).
