# Supplementary material for: Synthesis of clathrate cerium superhydride CeH9 at 80-100 GPa with atomic hydrogen sublattice
Source: Nat Commun. 2019 Oct 1;10:4453. doi: 10.1038/s41467-019-12326-y (PMC6773858; doi:10.1038/s41467-019-12326-y)
Supplement: Supplementary file 2 — Peer Review File [file 41467_2019_12326_MOESM2_ESM.pdf]

Editorial Note: This manuscript has been previously reviewed at another journal that is not operating a transparent peer review scheme. This document only contains reviewer comments and rebuttal letters for versions considered at *Nature Communications.*

Reviewers' comments:

Reviewer #1 (Remarks to the Author):

In the present version, the authors have addressed some points raised in my previous report.

However, I am still convinced that, although the work presented in the paper is of potential interest to a broad community,

because the synthesis of new superhydrides with large hydrogen content at relatively low pressures is very promising, the level of the discussion and of the theoretical analysis is not suited for a high-level publication.

In particular:

1) In the introduction, discussion, and even in the title of the paper, the authors overemphasize

the relation between the present compound and atomic hydrogen.

After five years of intense work in high-pressure hydrides, it is clear that, while

superhydrides may exhibit the large phonon frequencies and, in some cases, the

large electron-phonon matrix element that make atomic hydrogen attractive

for high-Tc superconductivity, they are actually extremely different from pure hydrogen,

and the nature and is not irrelevant.

In particular, while one may argue that having H-H distances in a reasonable range (larger

than molecular hydrogen, but smaller than in low-pressure metal hydrides) may be needed for high-Tc superconductivity,

it is also clear that having the same distance as in atomic hydrogen is totally irrelevant.

In fact, the data shown in figure 4 (c) show that there is no correlation whatsoever

between Tc and nearest H-H distance. FeH5, which is not superconducting, lies

between H3S and LaH10. LaH10, which has a record-high Tc, has longer distances than

CeH9, where the authors estimate a maximum Tc of 117 K.

I definitely don't see the added value of this discussion, and find it strongly misleading.

2) Superconductivity in another rare-earth polyhydride, LaH10, has been experimentally

measured and confirmed by two different groups.

The paper cites the first two reports, refs. 35 and 36, but not the most recent versions:

Drozdov et al., arXiv:1812.01561, M. Somayazulu et al., PRL 122, 027001 (2019).

which instead include a confirmation of high tc, and additional measurements that

characterize the superconducting phases.

These results are presented at a very late point of the discussion, and presented as

speculative, while I consider the evidence for superconductivity presented in these works

(magnetic field measurements, isotope effect, etc) much more convincing

than the empirical arguments on H-H distances presented in this paper.

A discussion of these experimental results, and of related theoretical works, is much more

compelling than the current focus of the discussion.

3) I stil have doubts on the first-principles calculations on CeH9, especially when compared

to similar calculations appeared in literature (ref. 21), and those contained in a

similar work submitted in parallel to Nat Chem. and now Nat. Comm. Apparently, at least for part of the calculations,

the authors of the two works used the same computational approach, the same code, the same crystal structure,

but obtained different results.

In particular, Ref. 21 reports that CeH9 in the P63/mmc structure is already stable

at 100 GPa, while the present paper reports the dynamical instability already at 150 GPa.

It is very likely that this sort of discrepancy comes from a strong phonon anharmonicity, combined with unconverged calculations.

Very anharmonic phonons are hard to converge at the harmonic level, especially with respect to integration over k space. Some information on the convergence tests done on this parameter, and on the level of confidence that one expects on the instability pressure, are required to assess the validity of the paper.

4) A question related to the previous one is that the authors describe in great detail a second

phase of CeH9, with C2/C symmetry, which is obtained from the high-pressure P63/mmc following the unstable eigenvectors.

Trying to identify this structure contradicts an argument proposed before, i.e. that the observed dynamical instability is not real, and that in experiments the P6/3mmc

phase is stabilized by quantum lattice effects.

If the stable structure is P6/3mmc, why should Tc calculated for a different structure

be relevant? Or do the authors imply that the experimental resolution is not enough to

discriminate between the two structures?

5) Coming back to the problem of comparing different sets of calculations, the other manuscript reports that the agreement

between for the lattice parameters between theory and experiments is improved in GGA+U compared to pure GGA.

It is again surprising that two calculations on the same systems, with the same method, obtain different results.

6) The manuscript reports Density of states, in which the f-derived states form

a narrow manyfold 2 eV above the Fermi level, with a long tail that extends down

to EF, so that f electrons give a substantial contribution to the DOS.

I think this is an artefact of using a pseudopotential that includes f electrons in valence, as it is also

well-known taht in DFT calculations the position in energy of the f states is wrong.

Therefore, any conclusion on the value and nature of the DOS at the Fermi level in such a system must be taken with care. What happens to the electronic structure, and in particular to the DOS shape,

when a realistic value of U is included in the calculation? How is the position of the f levels affected?

This is a more likely estimate of the "true" DOS of the system than the figures provided

in the manuscript.

7) Convergence of electron-phonon calculations: the authors have answered my previous

question about integration of electron-phonon matrix element in a non satisfactory way.

The authors have provided information about the q (phonon) grid used to calculate

the matrix elements, but do not describe the method using to perform the integration of the electron-phonon matrix elements over the Fermi surface. Which methods did the authors use?

What is the accuracy that the estimate on the value of lambda?

Errors in k space integration may lead to severe mistakes in the estimate of the electron-phonon coupling parameters, and hence of the superconducting Tc.

Convincing convergence tests in k space are needed to judge the accuracy of their Tc estimate.

Reviewers' comments:

Reviewer #1 (Remarks to the Author):

In the present version, the authors have addressed some points raised in my previous report. However, I am still convinced that, although the work presented in the paper is of potential interest to a broad community, because the synthesis of new superhydrides with large hydrogen content at relatively low pressures is very promising, the level of the discussion and of the theoretical analysis is not suited for a high-level publication.
In particular:

1) In the introduction, discussion, and even in the title of the paper, the authors overemphasize the relation between the present compound and atomic hydrogen. After five years of intense work in high-pressure hydrides, it is clear that, while superhydrides may exhibit the large phonon frequencies and, in some cases, the large electron-phonon matrix element that make atomic hydrogen attractive for high-Tc superconductivity, they are actually extremely different from pure hydrogen, and the nature and is not irrelevant.

In particular, while one may argue that having H-H distances in a reasonable range (larger than molecular hydrogen, but smaller than in low-pressure metal hydrides) may be needed for high-Tc superconductivity, it is also clear that having the same distance as in atomic hydrogen is totally irrelevant. In fact, the data shown in figure 4 (c) show that there is no correlation whatsoever between Tc and nearest H-H distance. FeH5, which is not superconducting, lies between H3S and LaH10. LaH10, which has a record-high Tc, has longer distances than CeH9, where the authors estimate a maximum Tc of 117 K. I definitely don't see the added value of this discussion, and find it strongly misleading.
Reply: We thank the reviewer for the comment. We think that both the hydrogenic sublattice and the nature of the metal atom are essential. The hydrogenic sublattice in CeH_9 approaches monatomic hydrogen closer than other hydrides, and this is only one factor.
To avoid misleading, we have added the following statement in our discussion,
- *". Judging from the H-H distances, CeH_9 is closer to monatomic metallic hydrogen than other hydrides, yet its predicted T_c is not as high as that of hydrogen or of LaH_{10}. This highlights the importance of the electronic structure of the metal atom, in agreement with the recent suggestion of Semenok et al[29]. In Fig. 6c, we can also see that non superconducting FeH_5 has shorter H-H distance than H_3S compound well known for very high T_c."*

2) Superconductivity in another rare-earth polyhydride, LaH10, has been experimentally measured and confirmed by two different groups. The paper cites the first two reports, refs. 35 and 36, but not the most recent versions: Drozdov et al., arXiv:1812.01561, M. Somayazulu et al., PRL 122, 027001 (2019). which instead include a confirmation of high tc, and additional measurements that characterize the superconducting phases. These results are presented at a very late point of the discussion, and presented as speculative, while I consider the evidence for superconductivity presented in these works (magnetic field measurements, isotope effect, etc) much more convincing than the empirical arguments on H-H distances presented in this paper.
A discussion of these experimental results, and of related theoretical works, is much more compelling than the current focus of the discussion.
Reply: We thank the reviewer for the comment. When we wrote this paper, the work of Somayazulu was unpublished. Now we have rearranged and included the discussion about the latest superconductivity reports of LaH_{10}, (*PRL 2019 and Nature 2019*) in our introduction.

We have added the following statements in the introduction section,

- *"Recently superconductivity with T_c of 260 K at 180 GPa and 250 K at 170 GPa were reported for LaH_{10} by two different research groups by electrical conductivity measurement[16,17]. Detecting Meissner effect to confirm the superconductivity was difficult because signal from extremely small sample is too weak to be picked up by the state-of-the-art techniques. At such pressure the verification of T_c becomes a challenging task. The latest study reported of T_c decreasing with the application of magnetic field and the observation of the effect of isotope on T_c support superconductivity in LaH_{10}[17]. The discovery of superconductivity in LaH_{10} is a milestone in the search of room temperature superconductivity."*

3) I still have doubts on the first-principles calculations on CeH9, especially when compared to similar calculations appeared in literature (ref. 21), and those contained in a similar work submitted in parallel to Nat Chem. and now Nat. Comm. Apparently, at least for part of the calculations, the authors of the two works used the same computational approach, the same code, the same crystal structure, but obtained different results. In particular, Ref. 21 reports that CeH9 in the P63/mmc structure is already stable at 100 GPa, while the present paper reports the dynamical instability already at 150 GPa.
It is very likely that this sort of discrepancy comes from a strong phonon anharmonicity, combined with unconverged calculations.
Very anharmonic phonons are hard to converge at the harmonic level, especially with respect to integration over k space. Some information on the convergence tests done on this parameter, and on the level of confidence that one expects on the instability pressure, are required to assess the validity of the paper.
Reply: Ref 21 (*Feng Peng et al PRL 2017*) only claims the stability of *P6₃/mmc*-CeH_9 at 200 GPa (other pressures were not explored), which is consistent with our manuscript. We found instability at and below 120 GPa. We included the temperature effect, capturing anharmonicity, and confirmed the dynamical stability of the *$P6_3/mmc$*-CeH_9 already at 100 GPa (see Fig R2).

Regarding the other slight difference between our work and the similar work submitted in parallel to Nature Communications, we checked, for example, the H..H bond distance and found that there is only ~0.01 Å difference, which is negligible.

We include this figure from Zhou et. al., *arXiv 2019*, which shows both our results along with the other work for the reviewer's notice.

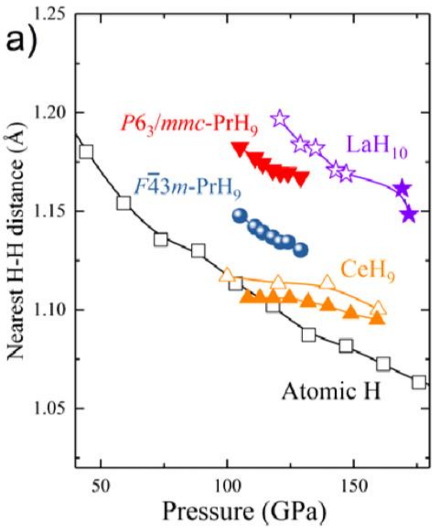

Figure R1: Comparison of the pressure dependence of the nearest H-H distances for hydrides, and atomic H was taken from Zhou et. al., *arXiv 2019*.

4) A question related to the previous one is that the authors describe in great detail a second phase of CeH9, with C2/C symmetry, which is obtained from the high-pressure P63/mmc following the unstable eigenvectors.
Trying to identify this structure contradicts an argument proposed before, i.e. that the observed dynamical instability is not real, and that in experiments the P6/3mmc phase is stabilized by quantum lattice effects.
If the stable structure is P6/3mmc, why should Tc calculated for a different structure be relevant? Or do the authors imply that the experimental resolution is not enough to discriminate between the two structures?
Reply: In order to calculate T_c for CeH_9 within the harmonic approximation at 100 GPa, we had to resolve the unstable eigenvectors, which resulted in symmetry breaking from $P6_3/mmc$ to *C2/c*.

From the experimental XRD point of view, these two structures, which indeed have a group-subgroup relationship, are indistinguishable. Similarly, instability of the $Fm\bar{3}m$-LaH_{10} is resolved by Geballe et al., *Angew. Chem. 2018* at 200 GPa and the stable configuration within harmonic approximation yields *C2/m* space group. It is worth mentioning that LaH_{10} shows instability within the harmonic approximation for the experimentally observed $Fm\bar{3}m$ phase at 200 GPa.

The high-symmetry structure is stabilized anharmonically. To include anharmonicity, we used the approach of [Hellman et al., *Phys. Rev. B 2011*], to obtain temperature dependent phonon frequencies and the phonon spectra show dynamical stability already at 100 GPa and 500 K (see Fig. R2).

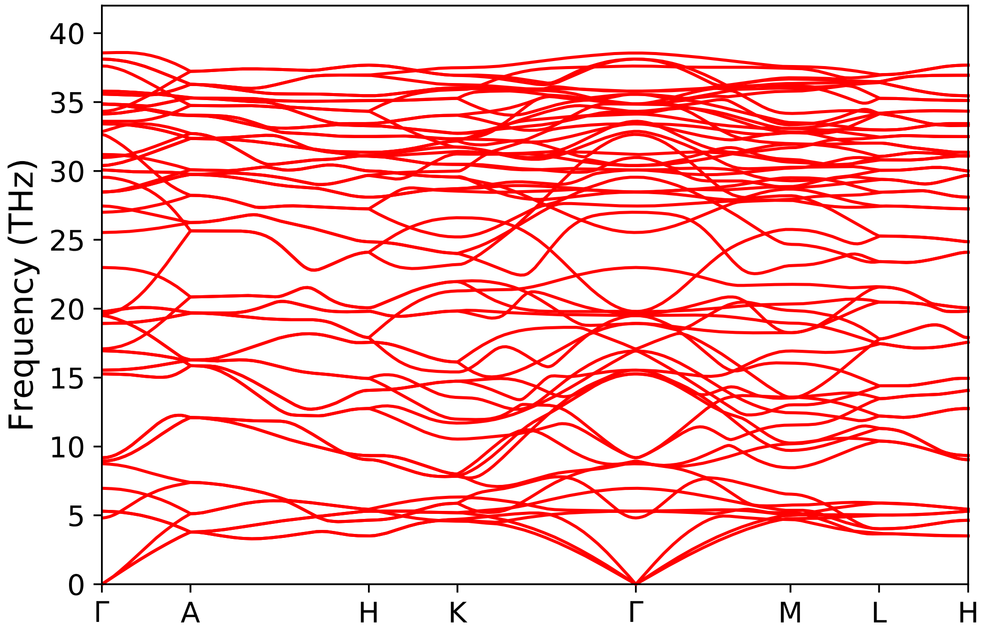

Figure R2: phonon dispersion curves for $P6_3/mmc$-CeH_9 at 100 GPa and 500 K. Absence of imaginary phonons in the dispersion curves shows the dynamical stability of $P6_3/mmc$-CeH_9 at 100 GPa and 500 K.

5) Coming back to the problem of comparing different sets of calculations, the other manuscript reports that the agreement between for the lattice parameters between theory and experiments is improved in GGA+U compared to pure GGA. It is again surprising that two calculations on the same systems, with the same method, obtain different results.
Reply: We thank the reviewer for the comments. We used Hubbard (DFT+U) corrections method and the results show that the PBE functional still gets the most accurate results

compared to the experimental values. We further checked GGA+U with different U values (U=4,5,6,7 eV) and LDA+U (U = 6 eV) to calculate cell parameters and the volume of the unit cell at different pressure conditions. Inclusion results in a slight change, while standard PBE agrees with experimental data better than the corrected approaches.

GGA+U gives a 0.6% difference in the calculation of cell parameters. Furthermore, it is now clear that calculations of T_c based on the GGA are rather accurate; whether or not the same is true for the GGA+U approach, is unclear. Therefore, it is a much safer approach to just use GGA.

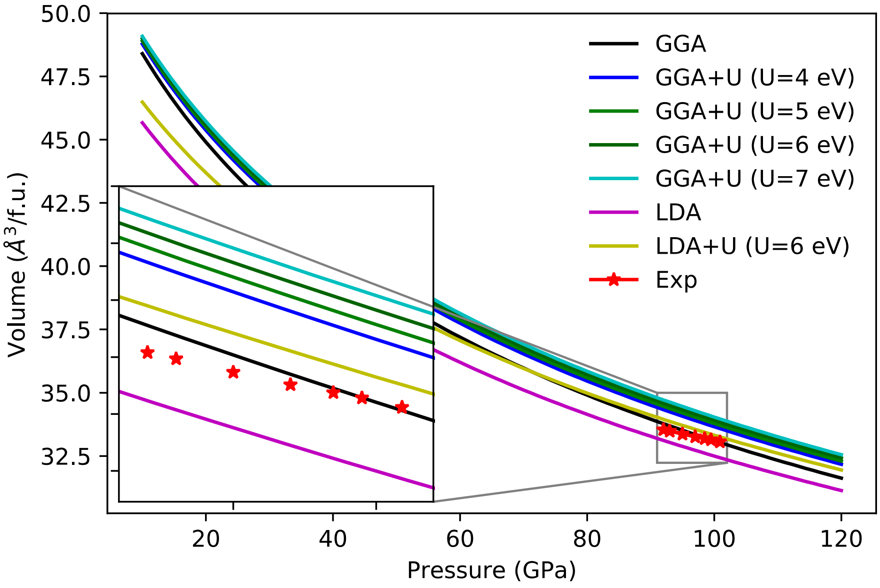

Figure R3: Comparison of Pressure-Volume EOS fit of various theoretical methods with experimental data.

6) The manuscript reports Density of states, in which the f-derived states form a narrow many-fold 2 eV above the Fermi level, with a long tail that extends down to Ef, so that f electrons give a substantial contribution to the DOS.
I think this is an artefact of using a pseudopotential that includes f electrons in valence, as it is also well-known that in DFT calculations the position in energy of the f states is wrong Therefore, any conclusion on the value and nature of the DOS at the Fermi level in such a system must be taken with care. What happens to the electronic structure, and in particular to the DOS shape, when a realistic value of U is included in the calculation? How is the position of the f levels affected?
This is a more likely estimate of the "true" DOS of the system than the figures provided in the manuscript.
Reply: We thank the reviewer for the comments. We agree with the reviewer that DFT calculates the position of f state incorrectly. However, strongly localized f-electrons do not couple to H-vibrations and do not affect T_c calculation. This can be seen from surprisingly low T_c values computed for hydrides of heavy rare earths in the literature. We included U values as 6 eV in the DOS calculations and added the corrected DOS values at the Fermi level in the manuscript. There is a marginal shift in the position of the f-levels and that results in lowering the values of DOS at the Fermi level.

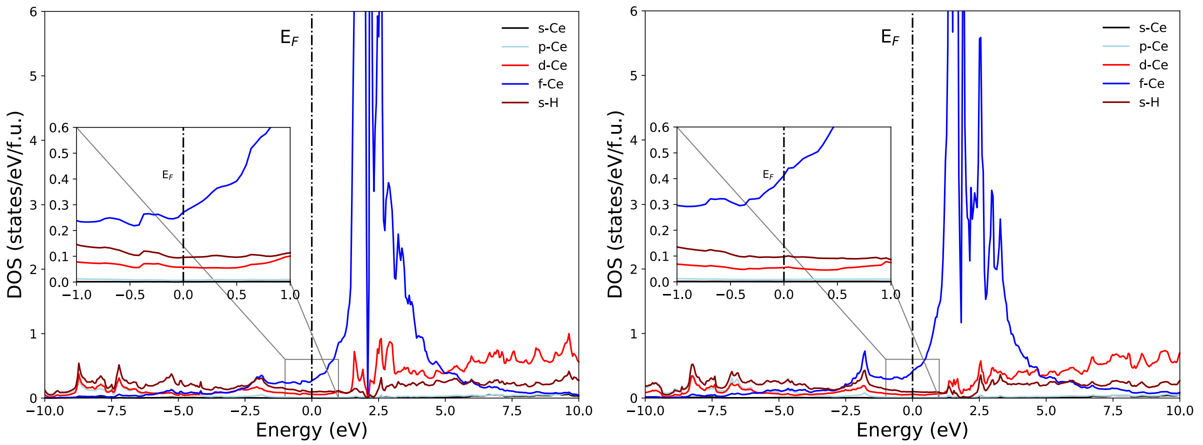

Figure R4: Electron density of states (DOS) for CeH_9 at 150 GPa (a) with (b) and without Hubbard correction. Electron DOS at the Fermi level is largely dominated by Ce-4f and H orbitals.

Table R1: Comparison of the electronic density of states of various hydrides. We included U = 6 eV (GGA+U) and the corrected DOS at the Fermi level are reported in the parenthesis.

**Hydride**	**Pressure (GPa)**	**N(Ef) (states/eV/f.u.)**
$P6_3/mmc$-CeH_9	100 GPa	0.92 (0.73)
$P6_3/mmc$-CeH_9	150 GPa	0.81 (0.67)
$P6_3/mmc$-CeH_9	200 GPa	0.73 (0.62)
$Im\bar{3}m$ -H_3S	200 GPa	0.52
$Fm\bar{3}m$ -LaH_{10}	200 GPa	0.73

7) Convergence of electron-phonon calculations: the authors have answered my previous question about integration of electron-phonon matrix element in a non-satisfactory way. The authors have provided information about the q (phonon) grid used to calculate the matrix elements, but do not describe the method using to perform the integration of the electron-phonon matrix elements over the Fermi surface. Which methods did the authors use? What is the accuracy that the estimate on the value of lambda? Errors in k space integration may lead to severe mistakes in the estimate of the electron-phonon coupling parameters, and hence of the superconducting Tc. Convincing convergence tests in k space are needed to judge the accuracy of their Tc estimate.

Reply: Electron-phonon is calculated by interpolation over the Brillouin Zone as in *Wierzbowska et al. arXiv:cond-mat/0504077*. We chose different Gaussian broadening in the electron-phonon coefficient calculations and 0.035 Ry gave a good convergence in the value of lambda (see Fig. R8). We also added the convergence of total energy with respect to k-points (Fig. R5), energy cutoff (Fig. R6), Methfessel-Paxton smearing width (Fig. R7) for the reviewer's notice. We have calculated and provided the electron-phonon coefficient (λ) values with respect to the k-point (Fig. R9). We also added to the manuscript, as well as here, T_c values estimated with different Coulomb pseudopotentials i.e., 0.10 and 0.15 which are widely accepted lower and upper bound values. This gives a range of critical temperatures for a given system, and better shows the uncertainty of T_c estimation.

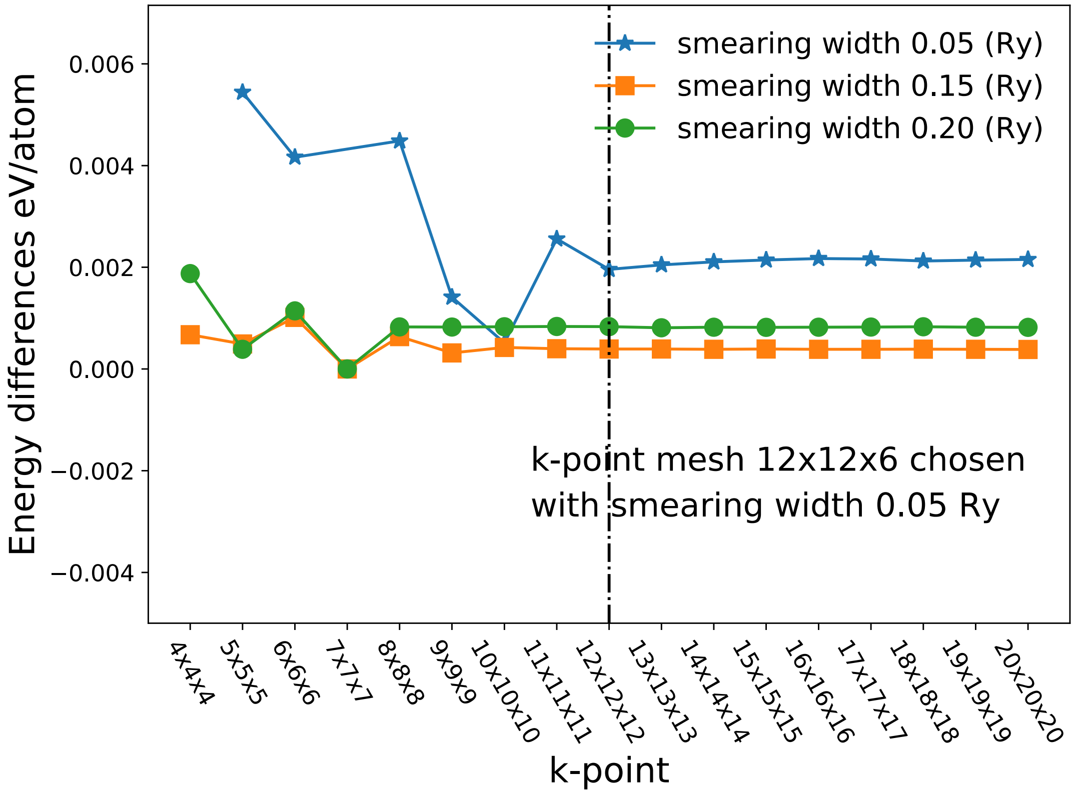

Figure R5

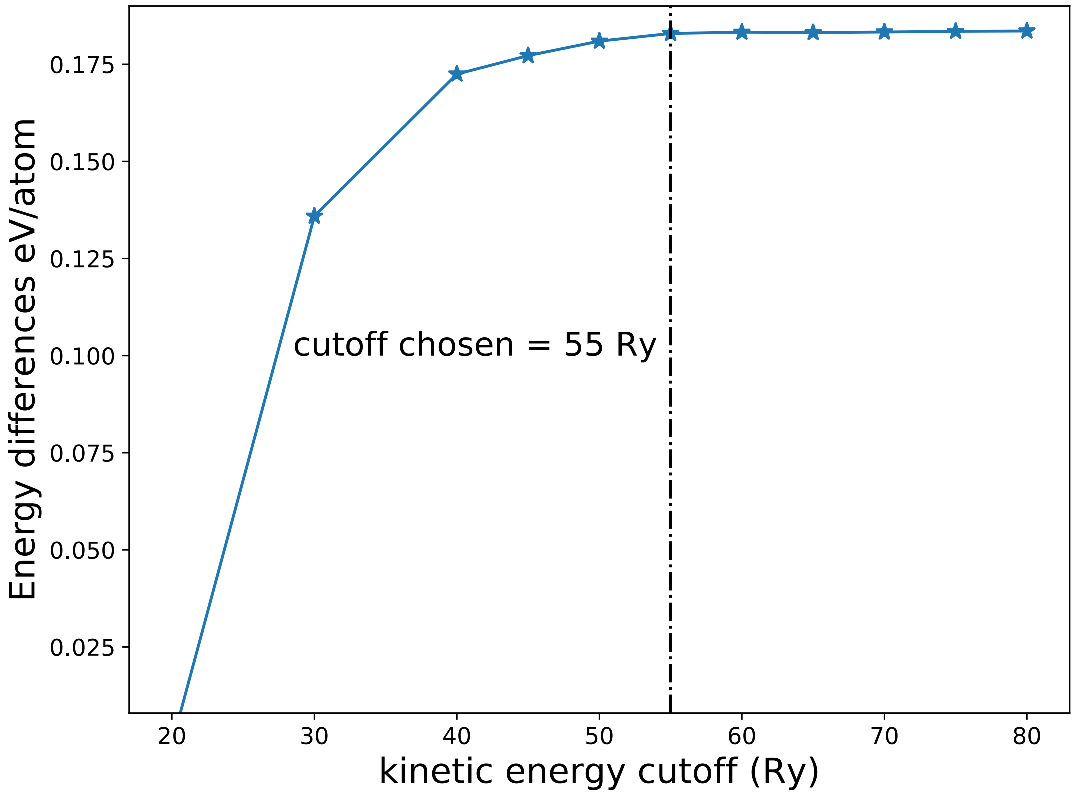

Figure R6

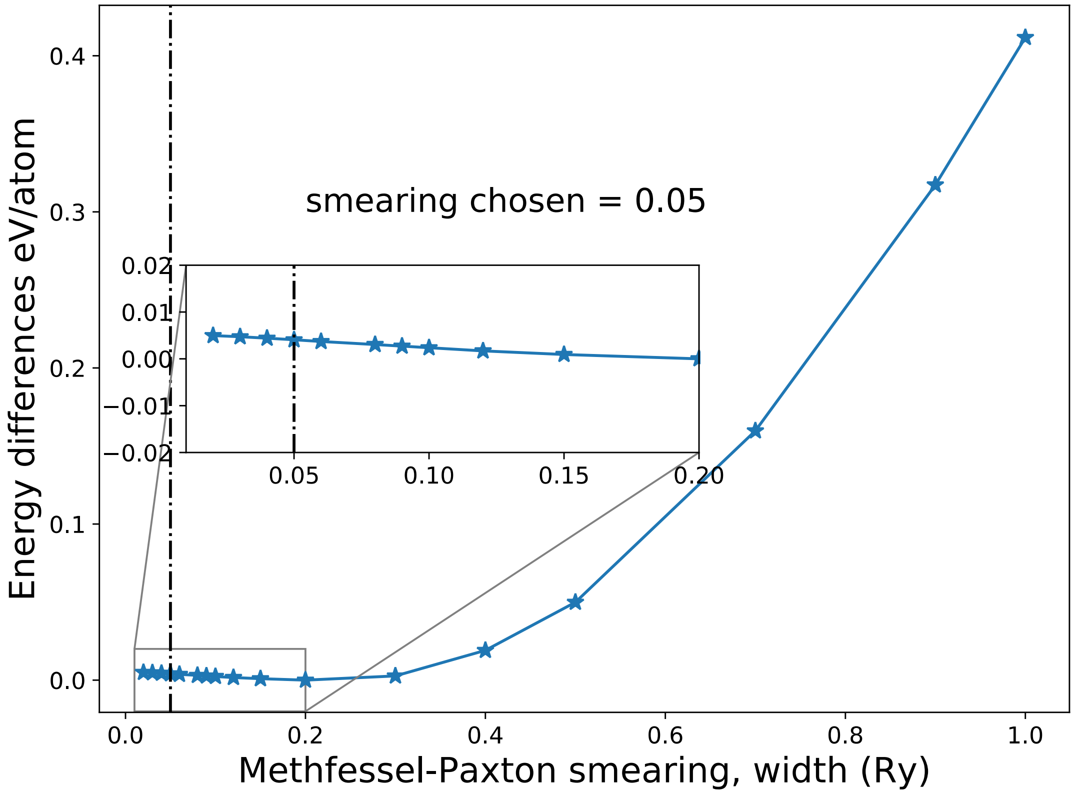

Figure R7

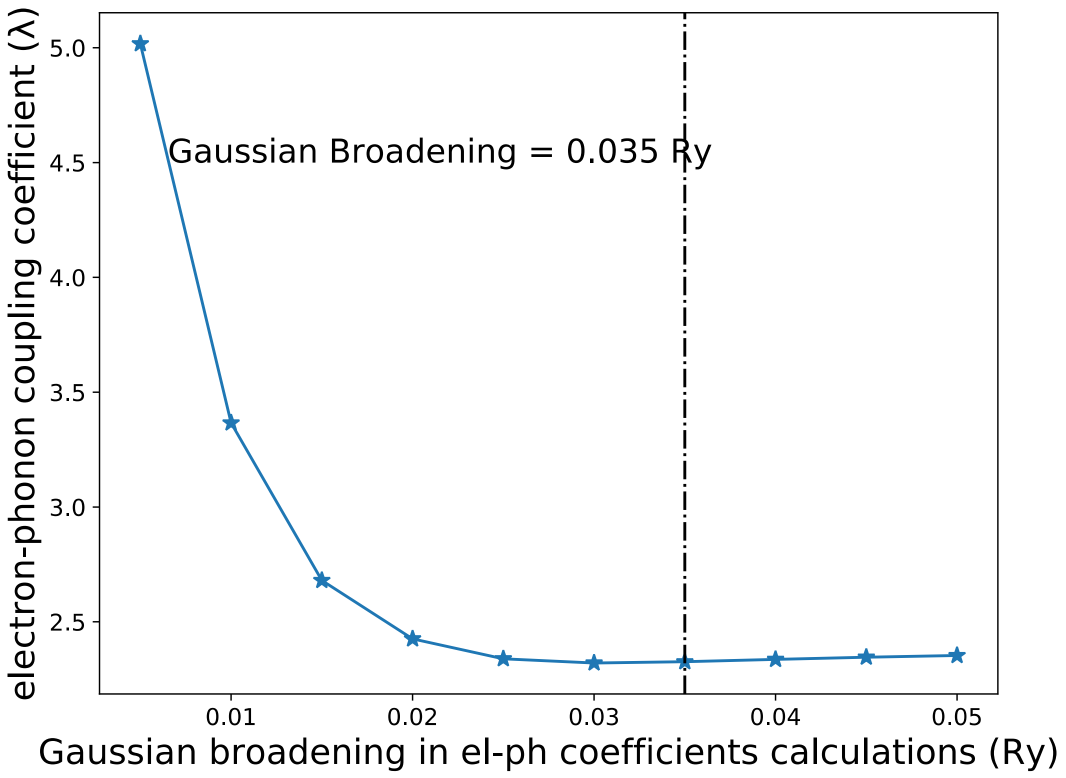

Figure R8

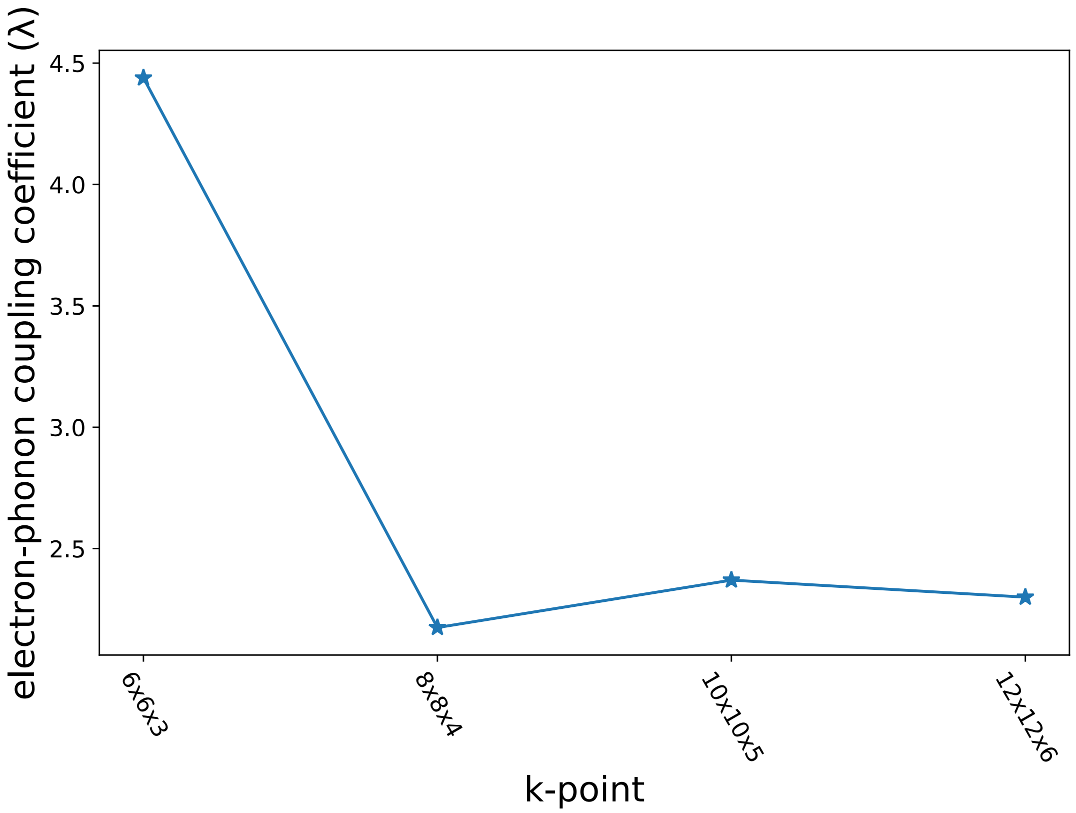

Figure R9

Table R2: Electron-phonon coupling coefficient (λ), logarithmic average phonon frequency (ω_{log}) and T_C for CeH_9.

Hydride	Space group	λ	ωlog (K)	*Tc* (K)	Pressure (GPa)
CeH_9	*P*6_3/*mmc*	2.30	740	117 (μ* = 0.10)<br>105 (μ* = 0.15)	200
CeH_9	*C*2/*c*	1.48	662	75 (μ* = 0.10)<br>63 (μ* = 0.15)	100

We also checked with various literatures and it appeared k-points grids with reciprocal space resolution of $2\pi \times 0.03$ Å^{-1} – $2\pi \times 0.05$ were employed in Brillouin zone sampling, in which gives good convergence in the calculated properties of various hydrides [1-4]. k-points grids with reciprocal space resolution of $2\pi \times 0.03$ Å^{-1} is chosen in our work.

1- Kvashnin, A. G.; Semenok, D. V.; Kruglov, I. A.; Wrona, I. A.; Oganov, A. R. High-Temperature Superconductivity in a Th–H System under Pressure Conditions. *ACS Appl. Mater. Interfaces* **2018**, *10* (50), 43809–43816.
2- Peng, F.; Sun, Y.; Pickard, C. J.; Needs, R. J.; Wu, Q.; Ma, Y. Hydrogen Clathrate Structures in Rare Earth Hydrides at High Pressures: Possible Route to Room-Temperature Superconductivity. *Phys. Rev. Lett.* **2017**, *119* (10), 107001.
3- Zhou, D.; Semenok, D. V.; Duan, D.; Xie, H.; Huang, X.; Chen, W.; Li, X.; Liu, B.; Oganov, A. R.; Cui, T. Superconducting Praseodymium Superhydrides. 23.

4- Geballe, Z. M.; Liu, H.; Mishra, A. K.; Ahart, M.; Somayazulu, M.; Meng, Y.; Baldini, M.; Hemley, R. J. Synthesis and Stability of Lanthanum Superhydrides. *Angew. Chem. Int. Ed.* **2018**, *57* (3), 688–692.

REVIEWERS' COMMENTS:

Reviewer #2 (Remarks to the Author):

The authors have successfully taken into account the points raised by Reviewer #1, by modifying and improving the paper accordingly, and by providing additional information which testifies the convergence of the calculations with respect to relevant parameters, such as the number of k-points, plane-wave cutoff, smearing. Therefore, I have no doubts that the calculations are carefully performed and analyzed. There is substantial agreement between the convex hull diagrams computed in PRL 119, 107001 (2017) and the ones obtained in the present paper. There is a small discrepancy though in the stability of the CeH8 phase at 100 GPa, stable in PRL119, unstable in the present work, but the energy difference between these two scenarios is below 30 meV/atom, a discrepancy that can be attributed to different convergence parameters in the two sets of calculations.

I agree with Reviewer #1 that the technique used to evaluate the electron-phonon coupling in the present paper is not the most up-to-date. Indeed, it is based on the interpolation method developed by Wierzbowska et al. in arXiv:cond-mat/0504077, while the most recent one is based on the Wannier functions interpolation. However, other approximations made here, such as the use of purely harmonic phonon spectrum, the treatment of correlation at the GGA-PBE level for the f-electrons, the overall correlation level taken into account by the Coulomb pseudopotential in the Eliashberg-McMillan approach, and the spin-orbit coupling neglected, are as crude as (or even more crude than) the interpolation method for the electron-phonon matrix elements. The estimate provided here for the superconducting critical temperature (Tc) of the CeH9 phase at 200 GPa must be interpreted in a semi-quantitative way in any case. It strongly indicates that also CeH9 can be a high-Tc BCS superconductor. I found this result very interesting, particularly because in this paper it is also shown that the clathrate CeH9 phase, bearing the highest Tc, has been synthesized. Therefore, the Tc reported here is a theoretical prediction which will certainly stimulate additional research, particularly from the experimental side. However, I recommend the authors to cite the arXiv paper by Wierzbowska et al. in the method section, when they explain which k-point grid has been used in the evaluation of the electron-phonon matrix elements.

There are discrepancies between the experimental and theoretical results presented in this paper. In particular, the pressure range for a stable Pm-3n-beta-UH3 phase is not reproduced precisely by the calculations, while in the high-pressure CeH9 region, the P6_3/mmc phase turns out to be stable only above 150 GPa in the calculations, while the experiment detects it already at 80 GPa. In the latter case, the authors provide evidence that the P6_3/mmc stabilization energy of CeH9 comes from anharmonic effects. However, the discrepancy between the Pm-3n-beta-UH3 and Pm-3n phases remains unexplained. I am wondering whether the authors could test the impact of the spin-orbit coupling (SOC), by including it in their calculations. The authors of Ref. arXiv:1904.06643 claim

that a more consistent phase diagram of the analogous system PrH_n is obtained with SOC inclusion, particularly in the low-pressure region of the convex hull diagram. The same should also be true in the Ce case, and it could account for the Pm-3n-beta-UH3/Pm-3n discrepancy. Please, check.

Regarding the high-pressure side of the phase diagram, where the clathrate CeH9 is stable, the one shown in Fig. 5 is not consistent with the more refined discussion presented later in the manuscript. Indeed, starting from the P6_3/mmc symmetry and following its dynamical instability in the 80-120 GPa pressure range, the authors found a stable, although less symmetric, C2/c phase. This implies that the C2/c phase has a lower energy than the P6_3/mmc phase. For some reasons, the C2/c phase has not been found in the USPEX crystal-search engine. However, this should be included in the general phase diagram of Fig. 7 for internal consistency, as it is a legitimate and well defined crystal structure.

Last but not least, I found several typos in the manuscript. A thorough proofreading of the paper is necessary.

The results presented in this manuscript are very interesting, timely and of potential high impact in the very active field of superhydrides. Therefore, I suggest its publication, provided the authors follow strictly my recommendations and suggestions.

REVIEWERS' COMMENTS:

Reviewer #2 (Remarks to the Author):

The authors have successfully taken into account the points raised by Reviewer #1, by modifying and improving the paper accordingly, and by providing additional information which testifies the convergence of the calculations with respect to relevant parameters, such as the number of k-points, plane-wave cutoff, smearing. Therefore, I have no doubts that the calculations are carefully performed and analyzed. There is substantial agreement between the convex hull diagrams computed in PRL 119, 107001 (2017) and the ones obtained in the present paper. There is a small discrepancy though in the stability of the CeH8 phase at 100 GPa, stable in PRL119, unstable in the present work, but the energy difference between these two scenarios is below 30 meV/atom, a discrepancy that can be attributed to different convergence parameters in the two sets of calculations.

I agree with Reviewer #1 that the technique used to evaluate the electron-phonon coupling in the present paper is not the most up-to-date. Indeed, it is based on the interpolation method developed by Wierzbowska et al. in arXiv:cond-mat/0504077, while the most recent one is based on the Wannier functions interpolation. However, other approximations made here, such as the use of purely harmonic phonon spectrum, the treatment of correlation at the GGA-PBE level for the f-electrons, the overall correlation level taken into account by the Coulomb pseudopotential in the Eliashberg-McMillan approach, and the spin-orbit coupling neglected, are as crude as (or even more crude than) the interpolation method for the electron-phonon matrix elements. The estimate provided here for the superconducting critical temperature (Tc) of the CeH9 phase at 200 GPa must be interpreted in a semi-quantitative way in any case. It strongly indicates that also CeH9 can be a high-Tc BCS superconductor. I found this result very interesting, particularly because in this paper it is also shown that the clathrate CeH9 phase, bearing the highest Tc, has been synthesized. Therefore, the Tc reported here is a theoretical prediction which will certainly stimulate additional research, particularly from the experimental side. However, I recommend the authors to cite the arXiv paper by Wierzbowska et al. in the method section, when they explain which k-point grid has been used in the evaluation of the electron-phonon matrix elements.
Reply: We thank the reviewer for the valuable comment. We already added the arXiv paper in the method section.

There are discrepancies between the experimental and theoretical results presented in this paper. In particular, the pressure range for a stable Pm-3n-beta-UH3 phase is not reproduced precisely by the calculations, while in the high-pressure CeH9 region, the P6_3/mmc phase turns out to be stable only above 150 GPa in the calculations, while the experiment detects it already at 80 GPa. In the latter case, the authors provide evidence that the P6_3/mmc stabilization energy of CeH9 comes from anharmonic effects. However, the discrepancy between the Pm-3n-beta-UH3 and Pm-3n phases remains unexplained. I am wondering whether the authors could test the impact of the spin-orbit coupling (SOC), by including it in their calculations. The authors of Ref. arXiv:1904.06643 claim that a more consistent phase diagram of the analogous system PrH_n is obtained with SOC inclusion, particularly in the low-pressure region of the convex hull diagram. The same should also be true in the Ce case, and it could account for the Pm-3n-beta-UH3/Pm-3n discrepancy. Please, check.
Reply: We have included spin-orbit coupling (SOC) and magnetism. SOC changes the enthalpy difference of CeH_3 phases by some 0.09 eV per atom. While this is significant,

experiment confirms the predicted phase, CeH_9. We also added a sentence in the manuscript about it in lines 143-146, as shown below.
- "*However, inclusion of spin-orbit coupling (SOC) and magnetism changes the enthalpy difference of* CeH_3 *phases by some 0.09 eV per atom. While these calculations affect predicted phase stability range, our experimental results confirm the existence of the predicted phase.*"

Regarding the high-pressure side of the phase diagram, where the clathrate CeH9 is stable, the one shown in Fig. 5 is not consistent with the more refined discussion presented later in the manuscript. Indeed, starting from the P6_3/mmc symmetry and following its dynamical instability in the 80-120 GPa pressure range, the authors found a stable, although less symmetric, C2/c phase. This implies that the C2/c phase has a lower energy than the P6_3/mmc phase. For some reasons, the C2/c phase has not been found in the USPEX crystal-search engine. However, this should be included in the general phase diagram of Fig. 7 for internal consistency, as it is a legitimate and well-defined crystal structure.
Reply: For the case of anharmonic systems, the Born-Oppenheimer energy surface may have some lower minimas with distorted structures. Calculations that include quantum effects with averaging atomic position in the distorted configurations would yeild a highly symmetic structure. This has been shown by Errea et al., arXiv:1907.11916 (2019) for anharmonic LaH_{10}, in which a low symmetry *C2* structure has a lower enthalpy than experimentaly synthesised phase. After inlcuding quantum effects, all low-symmetry phases collapse to a highly symmetric Fm-3m phase.

In CeH_9, we resolved the instablity within the harmonic approximation in a distorted structure with a lower symmetry *C2/c* which is a subgroup of $P6_3/mmc$ symmetry. *C2/c* has maximum of 2 meV/atom lower enthalpy than $P6_3/mmc$, but we believe the synthesized phase is of $P6_3/mmc$ symmetry and we already showed its dynamical stability with including temperature effect, capturing anharmonicity, at the synthesized pressure.

Last but not least, I found several typos in the manuscript. A thorough proofreading of the paper is necessary.
Reply: We apologize the for the typos. We have rectified all the typos.

The results presented in this manuscript are very interesting, timely and of potential high impact in the very active field of superhydrides. Therefore, I suggest its publication, provided the authors follow strictly my recommendations and suggestions.
Reply: We thank the reviewer for recommendation and suggestions.
